# Variability in Vowel Production within and between Days

**DOI:** 10.1371/journal.pone.0136791

**Published:** 2015-09-02

**Authors:** Shannon L. M. Heald, Howard C. Nusbaum

**Affiliations:** Department of Psychology, University of Chicago, Chicago, Illinois, United States of America; Birkbeck College, UNITED KINGDOM

## Abstract

Although the acoustic variability of speech is often described as a problem for phonetic recognition, there is little research examining acoustic-phonetic variability over time. We measured naturally occurring acoustic variability in speech production at nine specific time points (three per day over three days) to examine daily change in production as well as change across days for citation-form vowels. Productions of seven different vowels (/EE/, /IH/, /AH/, /UH/, /AE/, /OO/, /EH/) were recorded at 9AM, 3PM and 9PM over the course of each testing day on three different days, every other day, over a span of five days. Results indicate significant systematic change in F1 and F0 values over the course of a day for each of the seven vowels recorded, whereas F2 and F3 remained stable. Despite this systematic change within a day, however, talkers did not show significant changes in F0, F1, F2, and F3 between days, demonstrating that speakers are capable of producing vowels with great reliability over days without any extrinsic feedback besides their own auditory monitoring. The data show that in spite of substantial day-to-day variability in the specific listening and speaking experiences of these participants and thus exposure to different acoustic tokens of speech, there is a high degree of internal precision and consistency for the production of citation form vowels.

## Introduction

There is an enormous amount of acoustic to phonetic variability in the speech signal that arises from many sources. Peterson and Barney [1] conducted one of the first studies documenting this acoustic variability, demonstrating large, systematic between-talker acoustic variability among men, women and children as well as within-in talker acoustic variability among different tokens of vowels. Specifically, they found that across talkers the same acoustic pattern could denote different phonetic categories and further that for the same talker tokens of the same vowel could be acoustically distinct from each other even when the linguistic context is held the same. This latter finding by Peterson and Barney [1], that there is notable acoustic variability from token to token even within a talker, suggests that motor execution of articulation is not substantially regular.

Despite the tremendous amount of variation in the acoustic properties of phonetic categories, individuals rarely explicitly notice this variability and appear to effortlessly understand speech input as it was intended, although with some small performance penalty across variability in tokens (c.f. [2]). Early motor theories of speech perception (e.g., [3–4]) have suggested that understanding or modeling articulatory variability is critical to speech perception, as it is noise that must be filtered out by the listener. From a different perspective, Elman and McClelland [5] proposed that variability in speech results from systematicity in the processes that control speech production, and as such can be itself meaningful rather than just represent noise. If variability in speech results from systematicity in the processes that govern speech production, listeners may be able to separate the effects of such variability that acted in the production of a given speech signal to better recover the intended message. For variability to be informative however, it must be systematic so that it imparts aspects of the underlying information and physiological or anatomical structures that gave rise to the signal. As long as the variability of the signal in question is systematically exerted, listeners can use knowledge of the systematicity to correctly understand the intended signal. However, despite the potential informative nature of variability, the scientific characterization of the nature of acoustic variability in speech and its origins in articulation is far from complete. In particular, we do not know whether production variability can be described systematically for all aspects of variability. For example, while there is substantial research examining variability that arises due to co-articulation [6–9] and linguistic context (c.f. [10], for a review), very little work has examined the nature of acoustic variability within talkers over time.

Changes in fatigue [11–12], recent linguistic experience [13–14], affective state [15], and cognitive performance [16] over time can cause the acoustic pattern of a vowel to change. Further, early work on motor control for other motor systems shows that repeating a motor movement over time is associated with increases in movement output variability [17], which may be also true of speech motor movements. We know very little about these sources beyond knowing that they could contribute to within-speaker variability. Moreover, there is a general assumption that these sources of variability are simply random disturbances [18].

The goal of the current study is therefore to assess the nature of within talker variability both within and between days. Specifically, we examined such variability for citation form vowels, as they are generally considered to be the most fundamental part of the phonemic inventory [19]. This is because vowels are, by definition, a continuant sound, as there is no stoppage or occlusion of the airstream; as such, they are always produced with an open vowel tract. Additionally, the use of isolated vowels allowed us to eliminate and therefore control for additional variation related to consonant articulations (via co-articulation), which effects are known to be immense [3,6,20]. Any changes observed over time for citation-form, isolated vowels should be a consequence of a limited set of possible sources such as speaking experience, exposure to auditory signals, affective state, cognitive state or fatigue, which are known factors that affect the acoustic realization of vowels. In order to examine production over time participants were instructed to produce seven different vowels when prompted in random order—/EE/, /IH/, /AE/, /EH/, /AH/, /UH/, and /OO/ ten times each—at three different times over the span of a day- 9am, 3pm, 9pm–on each of three different days, every other day, over the course of 5 days. The spacing of the observation points was intended to give us a sampling of the day cycle for several days over the course of a week. We chose the vowels /EE/, /AE/, /OO/ and /AH/ because they are the four point vowels, as they represent the most extreme gestural positions in the vowel space. The other 3 vowels (/IH/, /EH/ and /UH/) were picked because they are clear non-point vowels and helped to balance the set to be a more even mix of tense verses lax vowels. Similar to Peterson and Barney [1], the fundamental frequency, first, second and third formants (F0, F1, F2, and F3 respectively) for each of the productions were measured. We asked two questions about the acoustic properties of these utterances: Does the mean frequency of specific acoustic properties (F0, F1, F2, or F3) change systematically or randomly across measurement sessions? Does the standard deviation of frequency for these properties (F0, F1, F2, or F3) change systematically across a session, indicating increased variability in production? We also measured the duration of each vowel, to see if a change in duration correlated with any F0 or spectra changes.

On one hand, an account of variability as noise or random variation predicts there should be no systematicity in any changes observed in the fundamental frequency, first, second and third formants (F0, F1, F2, and F3 respectively) over time. If there is a trend in an acoustic change in vowels between adjacent time points, a random noise model would predict reversals of these trends over time as regressions to the mean because any trends should be accidental. On the other hand, vowel target theories (e.g. [11,21–22]) would imply that any systematic change in F1 or F2 frequency values represents that the phonetic category information (i.e. the mental representation) guiding speech production has changed, even if slightly. Additionally, any systematic changes in standard deviation of formant frequency values suggests a change in phonetic precision in production or the control of articulation. If there is clear evidence of regular systematic change in vowel production over time, however, this is consistent with the premises of theories of speech perception that treat such variation as potential information for recognition processing rather than noise to be filtered out.

## Materials and Methods

### Participants

All eight participants (5 female) were University of Chicago students with native fluency in English who spoke with an Inland North dialect [23]. Each participant reported no history of speech or hearing disorders. All participants were between 18 and 30 years of age. Written consent was obtained from all participants before participation in the study. Upon completion of the study each participant was paid $40 for his or her participation. The Social and Behavioral Sciences Institutional Review Board of the University of Chicago approved this study (via IRB H05245), including all recruitment and experimental procedures.

### Stimuli and Design

During each testing session, we instructed speakers to produce isolated vowels associated with the following prompts displayed on a computer screen: AH as in hot, UH as in hut, IH as in hit, OO as in hoot, EH as in heck, AE as in hat and EE as in heat. As previously mentioned, these vowels were selected as they represent a fairly even split of point and non-point vowels, as well as tense and lax vowels. Speakers were instructed to say only the isolated vowel sound associated with each prompt. Speakers were given each prompt 10 times for each vowel category, for a total of 70 trials. Prompts were presented randomly. Each speaker was asked to come in three times a day (9am, 3pm, 9pm) for 3 visits evenly spaced over the course of five days. These times were chosen because we wanted to sample speech from throughout the waking day: speech recorded at 9am represented speech near the beginning of the waking day; speech recorded at 9pm signifies speech after a 12 hour day; and speech recorded at 3pm represents speech midway through a 12 hour day. Speech was collected every other day, as these days possessed similar time schedules for the participants. Before each session speakers said the isolated vowel sounds associated with each prompt once as practice. The order of these practice prompts was presented randomly. These practice trials were not included in the analysis. The speech was recorded on DAT tapes at a sampling rate of 48 kHz with a 16-bit resolution and the resulting tape was later digitized at a sampling rate of 44.1 kHz with a 16-bit resolution to accomplish analyses. Each sound was edited into its own separate sound file. Formant analysis was performed using Praat [24]. A selection window approximately 50 msec to 75 msec in length, taken from the middle twenty-five percent of each steady state isolated vowel, was used to estimate the first three formants using the Burg algorithm (as reported by Press et al. [25]). F0 analysis was also performed used Praat [24] using the autocorrelation method as described in [26]. The selection window was based on the pitch tracking function in Praat. The pitch floor was set to 75 Hz, the pitch ceiling to 600 Hz and the measurement interval’s time step was 10ms. For each vowel, the entire pitch tier was used to obtain the average F0. We defined the duration of the vowel as the length of its pitch tier in Praat and we measured this to the nearest 10 ms. Nightly sleep logs were kept to ensure that each participant had slept each night. As it is hard to imagine that men and women would show a different pattern in F0 or formant change over time, male and female F0 and formant frequency values were analyzed together.

The fundamental frequency, first, second and third formants (F0, F1, F2, and F3 respectively) for each of the productions were measured, as according to simple target models of perception, these formants represent the necessary and sufficient information to perceptually identify all vowels in the American English vowel space [27–28]. Systematic changes in formant values would suggest that the target for the phonetic category has changed (c.f. 11,21–22]). Additionally, systematic changes in standard deviation of formant frequency values would be indicative of changes in phonetic precision. While duration differences may be minimized for citation-form, isolated vowels, we nonetheless measured duration over the course of the day to see if there was a systematic change in duration of the course of day or across days that correlated with any F0 or spectra changes observed, especially since changes in speaking rate, perhaps due to fatigue, could result in changes in the acoustic realization of a vowel [29] and this might be signaled in duration changes.

## Results

Separate three-way repeated measures ANOVAs (Time point within a day x Days x Vowels) were performed for each of the following dependent measures: F0, F1, F2, F3, Standard Deviation of F0, Standard Deviation of F1, Standard Deviation of F2, Standard Deviation of F3 and Duration. Individuals’ average values for each of these dependent measures for each vowel at each time point can be found in following tables: S1 Table (Average F0), S2 Table (Average F1), S3 Table (Average F2), S4 Table (Average F3), S5 Table (Average Standard Deviation in F0), S6 Table (Average Standard Deviation in F1), S7 Table (Average Standard Deviation in F2), S8 Table (Average Standard Deviation in F3), and S9 Table (Average Duration).

**Fig 1:**
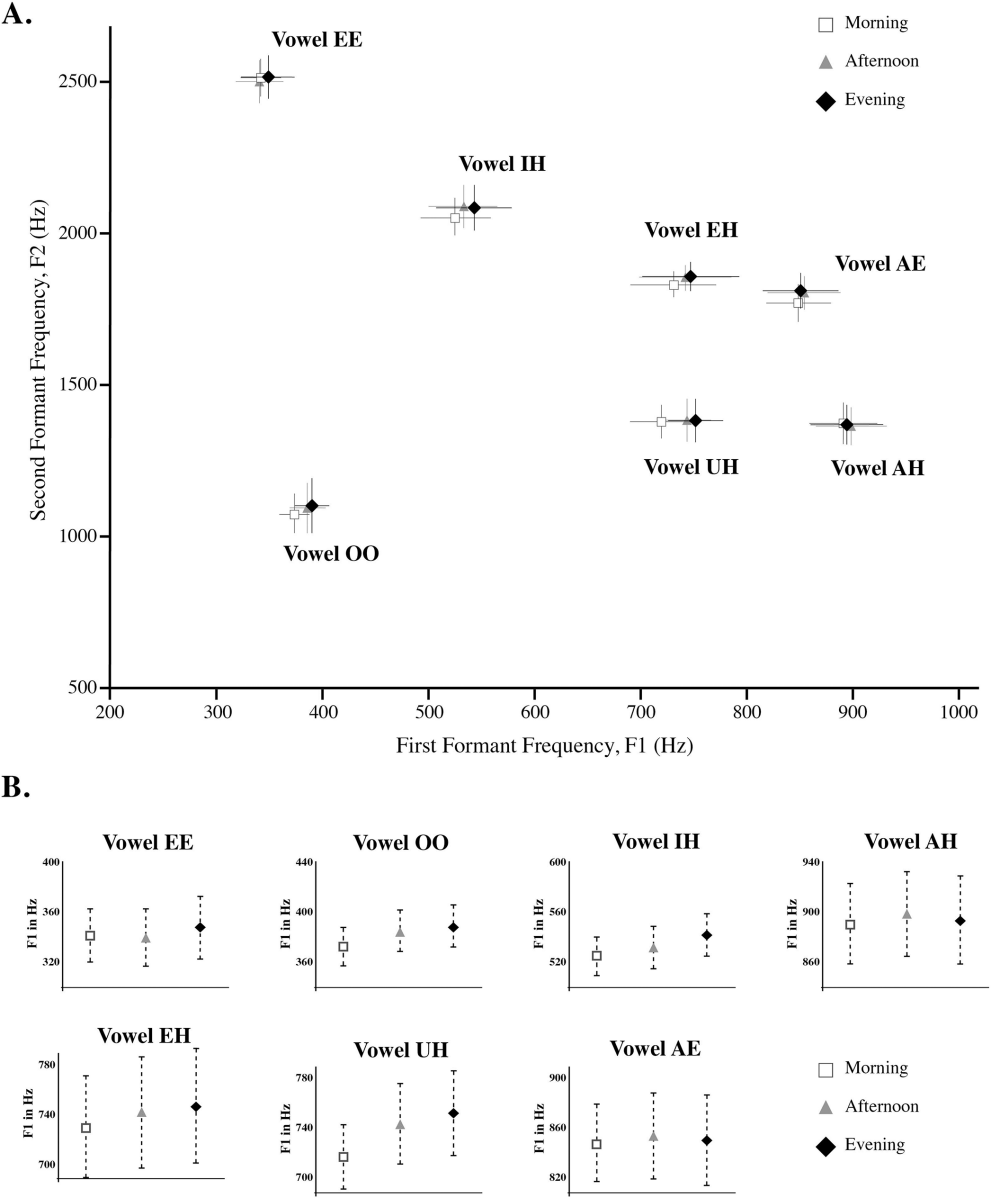
A. Mean F1 value by Mean F2 value plot at three different times (Morning, Afternoon and Evening) for each vowel tested across subjects. Error bars denote 1 standard error. B. Mean F1 values at three different times (Morning, Afternoon, and Evening) for each vowel tested across subjects. Error bars denote 1 standard error. As indicated by an average standard error of 27.60 Hz over the course day, there is high precision in isolated citation-form vowel production.

For F1, a significant systematic increase in mean frequency across all vowels over the course of a day was found (see Fig 1) such that F1 increased on average by 13 Hz [F1 Time point effect: F(2,14) = 4.39, p < .03]. While F1 increased over the course of a day, different vowels showed different sizes of F1 change, which is reflected in the significant interaction of Time point within a day x Vowel [F(12, 84) = 2.166, p < .021]. For vowels with naturally high F1 values (vowels /AH/ and /AE/), the increase of F1 was only 2.38 Hz with a standard deviation of 38.15 Hz whereas for naturally low F1 vowels (/EE/, /IH/, /EH/, /UH/ and /OO/) the increase of F1 was 17.66 Hz with a standard deviation of 34.36 Hz. Despite the different degree of shift in F1, the standard deviations are roughly comparable for the two types of vowels (38 Hz for high-F1 vowels and 34 Hz for low-F1 vowels). A post-hoc contrast between naturally high F1 vowels (/AH/ and /AE/) and naturally low F1 vowels (/EE/, /IH/, /EH/, /UH/ and /OO/) yields a significant difference for the mean shift in F1 within a day [t(79) = 2.411, p < .018]. In contrast to F1 values however, F2 values did not show a significant systematic change over the course of the day [F2 Time point effect: F(2,14) = .95, p < .41].

**Fig 2:**
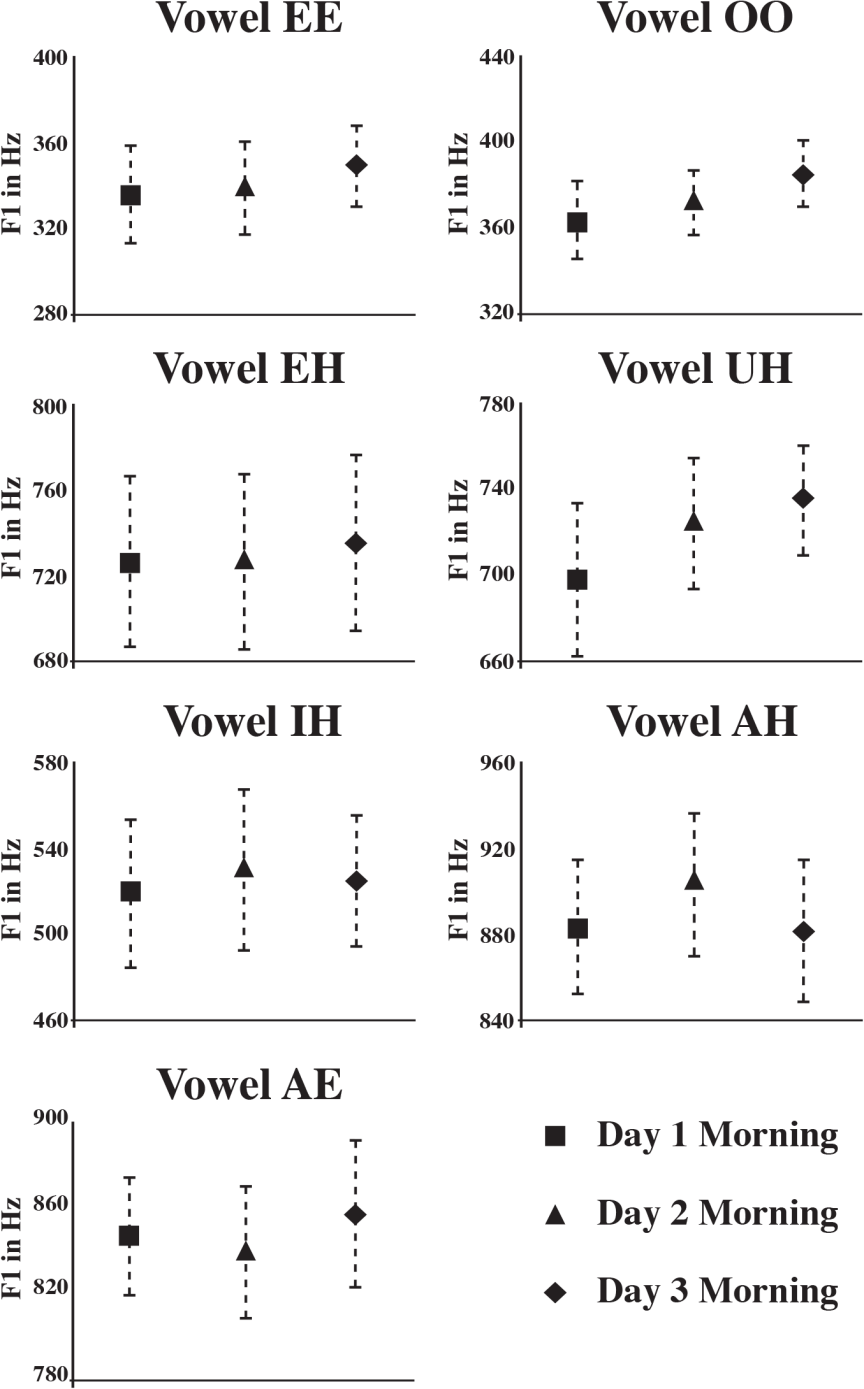
Morning F1 mean values across subjects with error bars showing standard error for each vowel for each day tested. As indicated by an average morning standard error of 29.96 Hz, there is high precision in isolated citation-form vowel production across mornings.

By contrast with the changes in production within a day, the difference between days was not significant for dependent measures F1 or F2 [F(2,14) = 1.92, p < .18 and F(2,14) = .67, p < .53 respectively]. It is important to note that the lack of a significant difference between days is not due to noise or increased variability compared to within a day. Both F1 and F2 vowel production was highly precise, with a standard error for F1 of 31.09 Hz across vowels and a standard error for F2 of 64.48 Hz across vowels (See Table 1, for the average F1 and F2 values in Hertz for each vowel for the three test days and the associated standard error). Moreover, despite the systematic increase over the course of the day in mean F1 values [F1 Time point within a day effect: F(2,14) = 4.39, p < .03], morning F1 values across days was quite reliable with a morning standard error across days of 29.96 Hz (See Fig 2, which plots the average morning F1 values and the associated standard error for each of the vowels tested). This suggests that sleep effectively resets F1 values for the following morning. The F1 values each morning are similar to each other but are systematically differ from F1 values at the end of the day. This means that changes occurring over the course of the day in F1 are eliminated by the next morning. This suggests that the restoration of morning F1 from evening F1 may well have happened during sleep. Sleep could simply offer a period of rest (auditorily or motorically) or it could be a period for consolidation [30–32].

**Table 1:** F1 and F2 average values and associated standard error (in Hz) for each vowel for the three days tested.

Vowel	Day	F1 in Hz (St. Error)	F2 in Hz (St. Error)
**AE**	**1**	848.74 (33.748)	1801.301 (55.05)
**AE**	**2**	847.624 (32.035)	1785.092 (58.835)
**AE**	**3**	855.225 (35.872)	1804.339 (69.111)
**AH**	**1**	888.876 (34.106)	1396.303 (62.624)
**AH**	**2**	898.628 (33.324)	1368.614 (67.994)
**AH**	**3**	894.236 (34.113)	1346.137 (69.287)
**EE**	**1**	336.558 (24.094)	2551.42 (64.665)
**EE**	**2**	343.017 (23.605)	2488.899 (57.945)
**EE**	**3**	349.187 (21.477)	2488.35 (87.477)
**EH**	**1**	738.531 (43.12)	1865.417 (56.621)
**EH**	**2**	735.015 (43.316)	1843 (33.929)
**EH**	**3**	744.734 (43.791)	1838.54 (43.754)
**IH**	**1**	529.602 (35.291)	2089.443 (77.148)
**IH**	**2**	533.456 (35.308)	2065.498 (53.183)
**IH**	**3**	535.675 (32.593)	2076.547 (83.045)
**OO**	**1**	369.12 (17.602)	1056.081 (56.582)
**OO**	**2**	392.923 (18.236)	1136.223 (110.635)
**OO**	**3**	385.556 (15.125)	1083.85 (81.243)
**UH**	**1**	726.6 (36.937)	1392.728 (55.943)
**UH**	**2**	737.705 (30.083)	1379.591 (50.763)
**UH**	**3**	748.173 (29.101)	1377.194 (58.354)

**Fig 3:**
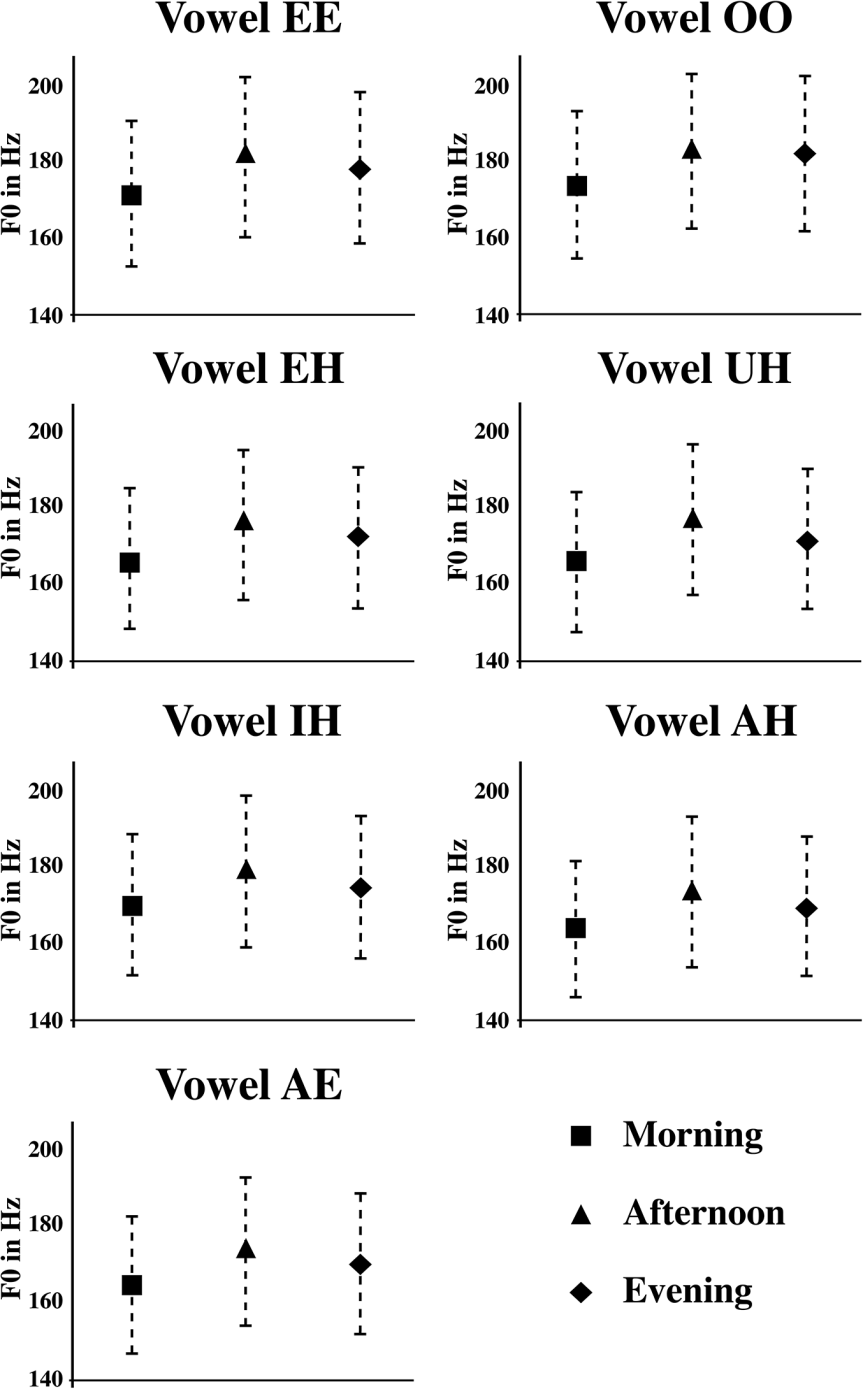
Mean F0 values at three different times (Morning (M), Afternoon (A) and Evening (E)) for each vowel tested across subjects. As indicated by an average standard error of 19.54 Hz over the course day, there is high precision in isolated citation-form vowel production.

A significant increase in F0 was also found over the course of a day, such that F0 increased by 9.42 Hz over the course of the day (See Fig 3) [F0 Time point within a day effect: F(2,14) = 6.79, p < .01]. An LSD post hoc contrast among the three time points (morning, afternoon and evening) yielded only a significant difference between the morning and afternoon sessions (Mean increase of 9.4 Hz, p < .001), the typical daytime nadir point in circadian rhythm for most of our participants [33–36]. This result is generally consistent with the rise in pitch induced by an increase in vocal productions (via fatigue or overuse) found in studies such as Gelfer et al. [37] and Stemple et al. [38].

It is important to point out that the systematic changes found in F1 and F0 over the course of a day are not solely being driven by lax vowels. This is a potential concerns as lax vowels may have been particularly novel targets in isolation, and therefore more prone to “learning” or “practice” effects. However, for F0, if the change over the course of the day was stronger for lax vowels (/IH/, /EH/, /AE/ and /UH/) than tense vowels (/EE/, /AH/ and /OO/), then we should have seen a significant Vowel by Time point within a day interaction effect for F0. This would have indicated that the change in F0 was significantly different for a subset of the vowels tested, but we do not find this. Further, while we do find a significant Vowel by Time point within a day interaction effect for the dependent measure F1, our post-hoc test reveals that all vowels show an increase in F1 over the course of the day except vowel /AE/ and vowel /AH/. This indicates that tense vowels, such as vowel /EE/ and vowel /OO/ additionally show the effect, which highlights that lax vowels alone are not responsible for the observed changes. Beyond this, we find no evidence that subjects had difficulty producing any of the vowels in isolation. In fact, we demonstrate quite the opposite: citation form vowels on a whole show incredible stability and reliability over the course of several days, with systematic changes only occurring within days, albeit slightly. In this sense the data highlight the extreme control of subjects to hold a stable posture for an intended vowel.

A significant main effect for Vowel was found for F0 [F(6,42) = 14.13, p < .001], F1 [F(6,42) = 170.40, p < .001], F2 [F(6,42) = 81.73, p < .001], F3 [F(6,42) = 14.36, p < .001], Standard deviation of F1 [F(6,42) = 4.48, p < .01], Standard deviation of F2 [F(6,42) = 2.46, p < .05] and duration [F(6,42) = 9.10, p < .001], indicating that the vowels differed among each other in F0, F1, F2, F3, and Standard deviation of F1 and F2. This is expected, as these measures are known cues for vowel identification. Along a similar vein, a significant main effect for Vowel was also found for the dependent measure of duration, indicating that some vowels types differ in intrinsic duration. This is likely due to our set of vowels including both tense and lax vowels. A paired sample t-test examining the duration differences between lax vowels (/IH/, /EH/, /AE/, and /UH/) and tense vowels (/EE/, /AH/, and /OO/) for each subject collapsed across days and time shows that lax vowels were significantly shorter than tense vowels [t(7) = -3.767, p < .01]. No significant change in duration was found over the course of a day [Main effect of Time point within a day for the dependent measure duration: F(2,14) = .014, p = .989] or across days [Main effect of Days for the dependent measure duration: F(2,14) = 1.229, p = .322]. Moreover, no interaction effects were found for the dependent measure duration.

No other significant effects or interactions were found for the dependent measures F3, standard deviation of F1, or the standard deviation of F2. Similarly there were no significant effects or interactions involving the Standard Deviation of F3, or the Standard Deviation of F0. An examination of the dependent measures via a BARK transform did not change any of these results.

## Discussion

The results demonstrate that citation-form speech production of isolated vowels is extremely precise and reliable. Further, the observed change in mean frequencies varies systematically over the course of a day, but not for all spectral features. In this sense the data highlight the extreme control of subjects to hold a stable posture for an intended vowel. The systematic change in F0 and F1 frequencies over the course of a day are systematic but still quite small, and thus unlikely to cause problems for listeners. Despite the systematicity of these changes, it is unlikely that listeners were aware of the change in F0 and F1 as they are well below the just noticeably difference (JND) in formant frequency discrimination, which is about 5% of the formant frequency [39]. However, if such variability were greater for non-citation form phonemes and therefore perceptible, listeners might in principle predict them. Under circumstances where such variability is systematic, listeners may be able to deconvolve the variability that acted on the speech signal to reveal the intended signal. In this sense, systematic and thusly predictable variation might be useful in perception (cf. [5]). These results mirror speech perturbation studies, which similarly demonstrate the excellent control that individuals possess in producing citation form vowels, as they rapidly compensate for perceived changes in F0, and spectra changes (e.g. [40]).

Moreover, the small but significant mean F1 frequency value change occurs without any change in variability overall, so the location of the vowel categories drifts slightly but reliably over time. Indeed, given that the F1 changes are comparable across the low F1 vowels (/EE/, /IH/, /EH/, /UH/, and /OO/), it reflects a slight change in the vowel space itself. This is different from changes in the size or shape of any particular category itself or any random change in the structure of the vowel space. To date, the current study is the first to provide empirical evidence suggesting that there is a small but reliable systematic change in speech production as a function of time of day.

It is unclear however, what is responsible for these systematic changes. We do not find any evidence that a change in duration underlie the systematic changes observed in F0 and F1 over the course of a day. However, there is considerable evidence that acoustic variability can occur within a talker due to a variety of other factors noted previously. For example, changes in fatigue [11–12], cognitive performance [16], affective state [15], local linguistic experience [13–14], ambient environment [41], and local word choice [42] can cause the acoustic realization of subsequent vowels to change over time. However, it is unclear if these or other factors contributed to the systematic changes in F1 and F0 over the course of the day because it is unclear that any of these factors would change this way over the course of a day.

Given that increased fatigue can affect formant and fundamental frequency values, and given that our observed changes occurred over the course of a day, one explanation of the present results might be that the talkers become fatigued later in the day due to prolonged vocal use over the course of the day. As reported by Kostyk & Rochet [43], a main symptom of vocal fatigue that arises from prolonged vocal use is the need to use greater vocal effort. This is because greater vocal effort is required to maintain loudness when fatigued. Traunmuller and Eriksson [44] reported that vocal effort (manipulated by physical distance between the talker and addressee) induces an increase to both F0 and F1, which is similar to the effects observed in the current study. Traunmuller and Eriksson speculate that vocal effort causes the larynx to rise, which can cause an increase in pitch. Additionally, Traunmuller and Eriksson warn that the rise in the larynx can additionally shorten the vocal tract causing the formants to rise, although they did not measure formant values in their data in evidence of this claim. In the current data however, we find an increase in both F0 and F1 over the course of the day. It is possible that change in vocal effort across the day caused the participants’ larynxes to rise, affecting both F0 and F1. However, in the current study, the F0 and F1 increase is only coupled in the morning and afternoon, as peak F0 values are found in the afternoon, whereas peak F1 values are found in the evening. Given that changes in formant values are not specifically coupled with changes in F0, it is unlikely that the increase in the first formant is solely due to voice quality changes engendered by changes in vocal effort. For example, it is possible, that changes in postural control of the head, neck and chest or arousal, may be responsible for the increase in F1. Gribble and Hertel [45] have shown that postural control decreases (causing their posture to become less rigid) over the course the day and that sleep restores this rigidity. Given that postural control is important for the proper planning and fine tuning of motor actions by providing reliable somatosensory information before, during, and after neuromuscular performance [46], decreased postural control throughout the day could have played a part in the observed increase in mean F1 values. If this is the case, the decrease in postural control may have cause subjects to inadequately monitor, and thusly poorly guide their speech apparatus [47]. In this sense, it is possible that the systematic changes found in the current experiment are reflective of other sources of variability other than vocal fatigue of the articulators.

Additionally, while there is ample evidence that linguistic experience can alter an individual’s phonetic perception and production [48–53], it is unlikely that the changes in F0 and F1 values resulted from any specific aspect of phonetic content, as each participant interacted with different people about different idiosyncratic topics over each day. Rather, if the F1 and F0 changes are indeed due to linguistic experience, it would be reflective of some kind of average or aggregate effect of using American English in the context of a mix of speakers at a university over the course of a day. For example, individuals listening to fatiguing talkers, who may not be fatigued from talking themselves, might acoustically converge to those around them along dimensions that are systematically changing. In this case, the early morning increase of F1 due to an increase in vocal effort may be perceived by listeners, and via a process of assimilation, cause them to alter their internal vowel target. This additional acoustic convergence of F1 would explain why F1 values become uncoupled from changes in F0 after the afternoon session, although further studies that directly examine the effect of linguistic experience on speech production are necessary.

The stability of F0, F1 and F2 across days is consistent with an interpretation based on vowel target theories assuming that the representation of vowel categories may be re-set by sleep in some fashion to maintain category stability. The systematic change in mean F0 and F1 values found in this study are not only restricted to within a day, but are resolved in order to instantiate a stable starting point in F0 and F1 values each day otherwise presumably F1 and F0 values would continue to further rise each day. Given the many hypothesized functions that sleep may provide (as a period for memory consolidation, synaptic pruning, restoration of vigor and restfulness, or simply as a passage of time without input), it is unclear what aspect of sleep is responsible for the re-setting of the F0 and F1 values observed across days.

The present results demonstrate remarkable stability in vowel production over the course of about a week. In citation form, in a controlled laboratory setting, speakers are capable of producing vowels with great reliability over days without any extrinsic feedback besides their own auditory monitoring. This stability is achieved in spite of natural and substantial variation in the linguistic experiences of these participants over this time period. Further, even given this stability in vowel production, there is clear evidence of some systematic changes in vowel production, both over the course of a day, and that vowel production is reset by the next day. All three of these observations—high precision, systematic within-day change in specific properties, and resetting of production on each day—are new information about speech production. The failure to show other kinds of changes in the present study coupled with the low variability indicates that in spite of varying language input and production, we still find small but highly systematic changes in citation form vowel production over time. Further work however will be necessary to understand the sources responsible for this variation and if such variation is present in co-articulated vowels found in fluent speech.

## Supporting Information

S1 Table Average F0 values (in Hz) for each vowel tested at each of the time points for each subject. (PDF)

S2 Table Average F1 values (in Hz) for each vowel tested at each of the time points for each subject. (PDF)

S3 Table Average F2 values (in Hz) for each vowel tested at each of the time points for each subject. (PDF)

S4 Table Average F3 values (in Hz) for each vowel tested at each of the time points for each subject. (PDF)

S5 Table Average Standard Deviation in F0 (in Hz) for each vowel tested at each of the time points for each subject. (PDF)

S6 Table Average Standard Deviation in F1 (in Hz) for each vowel tested at each of the time points for each subject. (PDF)

S7 Table Average Standard Deviation in F2 (in Hz) for each vowel tested at each of the time points for each subject. (PDF)

S8 Table Average Standard Deviation in F3 (in Hz) for each vowel tested at each of the time points for each subject. (PDF)

S9 Table Average duration (in ms) for each vowel tested at each of the time points for each subject. (PDF)
