# Supplementary material for: Variability in Vowel Production within and between Days
Source: PLoS One. 2015 Sep 2;10(9):e0136791. doi: 10.1371/journal.pone.0136791 (PMC4558024; doi:10.1371/journal.pone.0136791)
Supplement: S1 Table — (PDF) [file pone.0136791.s001.pdf]

Subject	Sex	Day	Time	Average F0 for /IH/	Average F0 for /EH/	Average F0 for /UH/	Average F0 for /EE/	Average F0 for /OO/	Average F0 for /AE/	Average F0 for /AH/
**1**	**Female**	**Day 1**	**9:00 AM**	170.84	174.82	173.20	189.20	202.39	167.82	163.48
**2**	**Female**	**Day 1**	**9:00 AM**	239.06	232.46	233.15	234.93	243.16	231.00	225.28
**3**	**Female**	**Day 1**	**9:00 AM**	180.96	178.13	175.58	179.60	184.46	170.77	171.85
**4**	**Female**	**Day 1**	**9:00 AM**	208.40	203.71	204.99	210.50	218.56	202.52	199.85
**5**	**Male**	**Day 1**	**9:00 AM**	101.18	95.44	98.75	97.83	103.55	92.05	92.14
**6**	**Male**	**Day 1**	**9:00 AM**	119.65	115.89	114.63	115.92	118.06	112.71	111.26
**7**	**Female**	**Day 1**	**9:00 AM**	169.44	171.33	170.49	177.77	183.07	169.99	166.30
**8**	**Male**	**Day 1**	**9:00 AM**	126.07	122.64	118.14	131.38	127.86	120.53	119.60
**1**	**Female**	**Day 1**	**3:00 PM**	253.16	250.18	252.37	255.39	258.01	249.36	251.23
**2**	**Female**	**Day 1**	**3:00 PM**	224.36	234.80	247.36	256.67	257.03	242.13	243.07
**3**	**Female**	**Day 1**	**3:00 PM**	199.04	191.05	193.30	198.12	209.06	183.66	182.29
**4**	**Female**	**Day 1**	**3:00 PM**	218.36	215.25	209.90	216.16	219.43	209.58	209.10
**5**	**Male**	**Day 1**	**3:00 PM**	103.84	98.20	96.79	102.46	104.65	98.07	93.36
**6**	**Male**	**Day 1**	**3:00 PM**	105.54	101.76	102.44	100.54	102.56	100.84	102.26
**7**	**Female**	**Day 1**	**3:00 PM**	185.76	175.07	176.37	185.81	195.33	169.95	170.63
**8**	**Male**	**Day 1**	**3:00 PM**	127.61	127.84	128.49	129.47	132.76	126.78	126.78
**1**	**Female**	**Day 1**	**9:00 PM**	203.81	201.98	220.86	221.03	222.41	209.46	206.50
**2**	**Female**	**Day 1**	**9:00 PM**	237.44	244.44	241.02	258.13	256.25	242.66	239.45
**3**	**Female**	**Day 1**	**9:00 PM**	198.60	192.29	194.24	201.62	209.32	186.52	185.98
**4**	**Female**	**Day 1**	**9:00 PM**	209.86	210.19	201.31	217.85	213.17	202.84	203.88
**5**	**Male**	**Day 1**	**9:00 PM**	108.45	104.03	102.49	107.79	106.73	102.47	99.38
**6**	**Male**	**Day 1**	**9:00 PM**	108.27	105.66	105.46	108.04	106.03	104.26	104.39
**7**	**Female**	**Day 1**	**9:00 PM**	185.99	180.32	177.90	199.30	205.24	177.31	174.95
**8**	**Male**	**Day 1**	**9:00 PM**	126.91	127.03	128.38	128.77	129.92	126.38	126.10
**1**	**Female**	**Day 2**	**9:00 AM**	264.97	261.77	261.65	265.87	268.24	262.17	261.99
**2**	**Female**	**Day 2**	**9:00 AM**	235.34	232.04	226.37	233.11	221.87	223.21	228.89
**3**	**Female**	**Day 2**	**9:00 AM**	189.75	182.63	176.29	183.51	183.83	172.51	172.45
**4**	**Female**	**Day 2**	**9:00 AM**	220.60	218.73	217.07	223.49	225.16	212.20	214.80
**5**	**Male**	**Day 2**	**9:00 AM**	104.80	104.12	103.15	105.59	106.82	103.95	97.98
**6**	**Male**	**Day 2**	**9:00 AM**	106.13	103.15	101.12	102.46	103.78	102.59	104.23
**7**	**Female**	**Day 2**	**9:00 AM**	159.91	152.16	149.96	168.48	164.37	154.87	148.79
**8**	**Male**	**Day 2**	**9:00 AM**	127.57	127.06	127.46	129.60	128.91	127.90	127.49
**1**	**Female**	**Day 2**	**3:00 PM**	224.32	221.39	217.93	227.75	222.97	219.85	221.00
**2**	**Female**	**Day 2**	**3:00 PM**	238.85	236.30	235.32	248.48	246.69	234.79	233.09
**3**	**Female**	**Day 2**	**3:00 PM**	200.72	195.74	193.60	195.33	204.95	182.41	181.20
**4**	**Female**	**Day 2**	**3:00 PM**	241.03	234.31	236.43	241.16	242.95	228.74	229.98
**5**	**Male**	**Day 2**	**3:00 PM**	106.19	105.88	105.55	106.50	106.18	104.64	97.95
**6**	**Male**	**Day 2**	**3:00 PM**	118.98	116.38	116.42	116.93	118.77	116.56	118.10
**7**	**Female**	**Day 2**	**3:00 PM**	179.04	174.12	173.59	191.08	191.13	174.36	170.31
**8**	**Male**	**Day 2**	**3:00 PM**	124.73	123.17	123.36	124.36	125.49	123.96	123.38
**1**	**Female**	**Day 2**	**9:00 PM**	221.71	218.53	220.15	221.93	221.85	214.87	221.01
**2**	**Female**	**Day 2**	**9:00 PM**	242.13	231.60	236.06	246.61	249.38	236.68	232.24
**3**	**Female**	**Day 2**	**9:00 PM**	208.73	204.79	199.55	211.42	225.44	192.73	194.77
**4**	**Female**	**Day 2**	**9:00 PM**	232.25	230.27	227.41	231.31	236.15	222.56	223.67
**5**	**Male**	**Day 2**	**9:00 PM**	104.56	103.10	102.06	103.82	106.26	99.66	96.11
**6**	**Male**	**Day 2**	**9:00 PM**	112.24	112.13	110.19	110.71	111.57	107.55	109.33
**7**	**Female**	**Day 2**	**9:00 PM**	181.35	175.72	173.94	186.48	194.94	176.66	175.91
**8**	**Male**	**Day 2**	**9:00 PM**	132.54	131.62	132.45	133.66	133.73	130.84	132.02
**1**	**Female**	**Day 3**	**9:00 AM**	239.21	236.72	236.02	240.91	241.87	231.29	234.44
**2**	**Female**	**Day 3**	**9:00 AM**	238.62	232.45	233.85	243.14	241.20	236.91	228.96
**3**	**Female**	**Day 3**	**9:00 AM**	193.37	190.23	182.35	188.97	195.45	175.21	174.70
**4**	**Female**	**Day 3**	**9:00 AM**	215.18	209.77	208.14	216.92	219.46	209.93	209.51
**5**	**Male**	**Day 3**	**9:00 AM**	96.21	92.58	92.15	99.49	96.97	97.50	96.85
**6**	**Male**	**Day 3**	**9:00 AM**	100.99	98.92	99.76	101.28	100.20	98.31	97.74
**7**	**Female**	**Day 3**	**9:00 AM**	160.55	150.56	152.17	169.76	176.14	159.12	155.94
**8**	**Male**	**Day 3**	**9:00 AM**	130.26	128.66	129.11	130.65	131.10	129.62	129.87
**1**	**Female**	**Day 3**	**3:00 PM**	248.29	246.70	246.70	248.63	252.21	244.91	247.84
**2**	**Female**	**Day 3**	**3:00 PM**	246.62	243.21	244.04	251.71	253.45	241.93	239.32
**3**	**Female**	**Day 3**	**3:00 PM**	201.89	193.23	197.03	210.02	211.97	188.63	185.22
**4**	**Female**	**Day 3**	**3:00 PM**	234.08	226.17	224.24	233.36	204.54	222.20	222.46
**5**	**Male**	**Day 3**	**3:00 PM**	103.59	97.75	99.98	102.95	105.06	101.05	100.31
**6**	**Male**	**Day 3**	**3:00 PM**	116.27	117.45	118.39	118.74	118.34	114.28	116.68
**7**	**Female**	**Day 3**	**3:00 PM**	174.45	170.84	164.92	175.02	183.89	166.77	169.93
**8**	**Male**	**Day 3**	**3:00 PM**	134.50	134.57	135.55	136.45	135.40	134.56	134.45
**1**	**Female**	**Day 3**	**9:00 PM**	226.80	224.60	224.96	231.62	226.18	224.59	223.38
**2**	**Female**	**Day 3**	**9:00 PM**	236.82	239.75	224.13	246.76	248.33	239.38	232.80
**3**	**Female**	**Day 3**	**9:00 PM**	203.78	197.26	193.27	200.06	220.34	189.81	181.92
**4**	**Female**	**Day 3**	**9:00 PM**	220.61	214.34	218.11	224.94	231.21	213.35	213.72
**5**	**Male**	**Day 3**	**9:00 PM**	99.31	97.70	97.49	97.48	102.23	99.42	96.67
**6**	**Male**	**Day 3**	**9:00 PM**	110.59	109.89	109.50	113.21	114.13	109.84	112.51
**7**	**Female**	**Day 3**	**9:00 PM**	175.05	168.86	170.06	179.11	187.61	170.90	168.85
**8**	**Male**	**Day 3**	**9:00 PM**	122.55	121.24	121.60	123.63	124.91	121.98	123.26
