# Supplementary material for: Variability in Vowel Production within and between Days
Source: PLoS One. 2015 Sep 2;10(9):e0136791. doi: 10.1371/journal.pone.0136791 (PMC4558024; doi:10.1371/journal.pone.0136791)
Supplement: S2 Table — (PDF) [file pone.0136791.s002.pdf]

Subject	Sex	Day	Time	Average F1 for /IH/	Average F1 for /EH/	Average F1 for /UH/	Average F1 for /EE/	Average F1 for /OO/	Average F1 for /AE/	Average F1 for /AH/
1	Female	Day 1	9:00 AM	629.19	749.74	766.76	354.03	382.52	834.11	883.05
2	Female	Day 1	9:00 AM	650.67	902.75	864.30	437.06	442.81	919.66	1069.33
3	Female	Day 1	9:00 AM	530.59	828.20	717.16	355.07	352.73	875.23	914.37
4	Female	Day 1	9:00 AM	597.70	790.68	765.84	401.06	426.09	899.93	859.00
5	Male	Day 1	9:00 AM	377.52	572.11	631.71	241.22	325.91	753.77	833.20
6	Male	Day 1	9:00 AM	449.15	620.16	572.72	287.41	328.11	765.65	858.02
7	Female	Day 1	9:00 AM	473.30	728.23	688.75	349.25	360.29	961.97	903.14
8	Male	Day 1	9:00 AM	446.35	624.77	573.52	263.95	288.98	766.72	751.29
1	Female	Day 1	3:00 PM	625.17	789.59	801.96	344.82	393.99	862.39	922.15
2	Female	Day 1	3:00 PM	665.63	890.79	902.26	430.86	440.39	910.32	1034.24
3	Female	Day 1	3:00 PM	598.14	884.61	807.31	377.94	361.41	919.96	985.33
4	Female	Day 1	3:00 PM	562.54	758.45	742.75	392.02	421.69	899.57	814.09
5	Male	Day 1	3:00 PM	387.36	567.65	705.75	246.14	351.75	740.06	874.80
6	Male	Day 1	3:00 PM	480.47	639.17	609.44	274.09	341.82	790.38	863.81
7	Female	Day 1	3:00 PM	495.11	811.81	753.39	346.91	376.82	1009.88	909.42
8	Male	Day 1	3:00 PM	423.19	597.90	572.66	266.18	280.97	701.78	704.20
1	Female	Day 1	9:00 PM	617.39	769.46	768.71	366.64	403.66	828.83	918.75
2	Female	Day 1	9:00 PM	695.76	889.57	905.50	438.57	456.80	924.63	1063.91
3	Female	Day 1	9:00 PM	640.41	904.85	851.78	379.98	376.23	934.50	971.64
4	Female	Day 1	9:00 PM	548.22	764.71	768.35	396.43	403.81	897.61	855.24
5	Male	Day 1	9:00 PM	371.41	579.10	699.46	240.64	365.04	725.33	870.52
6	Male	Day 1	9:00 PM	467.83	644.70	636.73	245.77	303.26	762.03	837.78
7	Female	Day 1	9:00 PM	524.80	834.48	767.91	352.85	367.57	987.36	921.86
8	Male	Day 1	9:00 PM	452.54	581.27	563.66	288.51	306.22	698.08	713.85
1	Female	Day 2	9:00 AM	611.72	759.38	793.54	313.60	363.51	840.91	973.92
2	Female	Day 2	9:00 AM	702.03	840.39	825.10	448.35	441.52	892.70	1060.38
3	Female	Day 2	9:00 AM	615.05	888.87	821.25	366.24	347.48	918.03	960.73
4	Female	Day 2	9:00 AM	527.47	755.06	739.58	404.25	427.13	914.03	856.73
5	Male	Day 2	9:00 AM	371.84	566.10	628.36	277.43	393.09	712.68	829.01
6	Male	Day 2	9:00 AM	482.56	644.74	676.70	277.81	321.90	788.70	910.15
7	Female	Day 2	9:00 AM	494.29	774.56	714.91	337.79	336.11	928.02	900.45
8	Male	Day 2	9:00 AM	436.78	588.12	589.66	285.92	336.82	722.45	747.81
1	Female	Day 2	3:00 PM	612.24	757.33	747.19	355.71	415.84	846.30	900.62
2	Female	Day 2	3:00 PM	679.81	851.93	871.16	465.07	459.00	927.98	1051.82
3	Female	Day 2	3:00 PM	603.47	923.62	852.13	358.44	373.58	939.79	984.07
4	Female	Day 2	3:00 PM	521.76	740.19	756.69	391.84	445.24	866.28	841.81
5	Male	Day 2	3:00 PM	378.84	571.75	731.80	239.89	456.34	735.45	885.91
6	Male	Day 2	3:00 PM	474.08	629.23	640.13	283.51	334.73	761.71	885.71
7	Female	Day 2	3:00 PM	487.75	772.02	700.21	317.12	361.59	946.89	919.11
8	Male	Day 2	3:00 PM	476.23	596.62	633.16	306.71	339.27	734.14	732.76
1	Female	Day 2	9:00 PM	620.62	775.09	763.91	354.05	422.52	861.73	919.92
2	Female	Day 2	9:00 PM	681.08	866.84	852.67	457.87	456.62	892.61	1046.64
3	Female	Day 2	9:00 PM	627.88	928.28	870.28	406.28	414.38	960.92	991.53
4	Female	Day 2	9:00 PM	540.60	805.01	765.02	400.29	433.78	927.89	864.00
5	Male	Day 2	9:00 PM	402.45	594.67	692.14	259.74	520.73	740.56	889.19
6	Male	Day 2	9:00 PM	469.85	626.12	654.49	273.99	318.39	798.06	866.99
7	Female	Day 2	9:00 PM	536.05	791.50	766.10	369.77	373.62	964.38	830.97
8	Male	Day 2	9:00 PM	448.51	592.93	618.75	280.76	336.94	720.76	716.86
1	Female	Day 3	9:00 AM	594.29	749.00	742.69	345.59	380.58	858.08	936.73
2	Female	Day 3	9:00 AM	661.38	854.07	821.73	453.37	451.75	910.67	1024.73
3	Female	Day 3	9:00 AM	606.85	915.33	837.13	371.68	368.95	935.16	965.54
4	Female	Day 3	9:00 AM	518.08	763.54	756.83	388.58	412.16	959.51	822.69
5	Male	Day 3	9:00 AM	398.27	600.03	709.21	311.93	442.50	723.98	881.46
6	Male	Day 3	9:00 AM	468.20	631.77	667.97	294.02	345.24	800.96	841.56
7	Female	Day 3	9:00 AM	492.96	774.02	723.55	331.34	352.44	952.87	864.08
8	Male	Day 3	9:00 AM	462.01	600.67	618.91	298.58	326.91	720.38	720.72
1	Female	Day 3	3:00 PM	620.40	729.34	726.56	357.23	427.91	853.77	950.90
2	Female	Day 3	3:00 PM	638.21	869.62	830.55	452.87	463.91	894.03	1005.95
3	Female	Day 3	3:00 PM	636.61	950.51	906.26	378.10	387.02	992.31	1002.83
4	Female	Day 3	3:00 PM	523.65	766.49	770.99	382.51	424.79	916.53	881.50
5	Male	Day 3	3:00 PM	393.68	614.78	724.72	263.26	384.81	720.40	919.11
6	Male	Day 3	3:00 PM	484.92	659.63	699.44	276.82	323.31	864.79	941.32
7	Female	Day 3	3:00 PM	524.24	826.77	729.05	349.33	351.01	955.26	832.06
8	Male	Day 3	3:00 PM	469.44	591.86	608.26	299.40	330.42	692.08	711.69
1	Female	Day 3	9:00 PM	615.14	761.00	753.41	356.23	414.34	856.74	930.90
2	Female	Day 3	9:00 PM	720.58	892.38	883.28	450.40	441.53	910.11	999.21
3	Female	Day 3	9:00 PM	605.07	932.46	870.67	381.88	379.49	944.43	989.60
4	Female	Day 3	9:00 PM	535.09	733.21	767.91	407.46	416.85	892.81	854.32
5	Male	Day 3	9:00 PM	390.32	585.31	698.90	266.04	377.43	705.35	889.48
6	Male	Day 3	9:00 PM	487.41	661.32	720.93	266.48	319.67	824.49	979.25
7	Female	Day 3	9:00 PM	548.20	816.51	776.50	404.09	394.29	958.58	838.45
8	Male	Day 3	9:00 PM	461.22	594.00	610.71	293.29	336.04	682.12	677.58
