# Supplementary material for: Variability in Vowel Production within and between Days
Source: PLoS One. 2015 Sep 2;10(9):e0136791. doi: 10.1371/journal.pone.0136791 (PMC4558024; doi:10.1371/journal.pone.0136791)
Supplement: S3 Table — (PDF) [file pone.0136791.s003.pdf]

Subject	Sex	Day	Time	Average F2 for /IH/	Average F2 for /EH/	Average F2 for /UH/	Average F2 for /EE/	Average F2 for /OO/	Average F2 for /AE/	Average F2 for /AH/
1	Female	Day 1	9:00 AM	2077.26	1860.58	1461.00	2763.04	1045.20	1827.90	1505.81
2	Female	Day 1	9:00 AM	2139.96	1868.26	1735.96	2566.46	1250.84	1864.47	1810.18
3	Female	Day 1	9:00 AM	2115.95	1834.23	1362.16	2761.19	1132.82	1737.17	1420.55
4	Female	Day 1	9:00 AM	2036.06	1824.62	1327.61	2491.44	1211.25	1857.08	1183.92
5	Male	Day 1	9:00 AM	2297.21	2095.69	1248.57	2461.75	1038.86	1981.80	1271.33
6	Male	Day 1	9:00 AM	1924.71	1778.84	1273.64	2361.07	1013.92	1750.04	1315.06
7	Female	Day 1	9:00 AM	2414.19	2150.18	1437.79	2809.36	846.64	1958.82	1358.49
8	Male	Day 1	9:00 AM	1833.08	1709.98	1247.18	2251.46	926.70	1566.75	1233.38
1	Female	Day 1	3:00 PM	2085.29	1660.94	1468.95	2584.99	1085.43	1487.74	1520.91
2	Female	Day 1	3:00 PM	1887.28	1669.84	1720.55	2291.03	1238.19	1619.68	1666.88
3	Female	Day 1	3:00 PM	2233.03	1895.21	1458.66	2795.50	987.28	1813.98	1486.00
4	Female	Day 1	3:00 PM	1639.77	1767.92	1327.64	2566.94	1251.37	1860.21	1160.71
5	Male	Day 1	3:00 PM	2346.89	2158.58	1241.79	2509.30	1173.90	2074.97	1365.88
6	Male	Day 1	3:00 PM	1921.04	1831.00	1224.58	2412.60	960.70	1679.73	1259.05
7	Female	Day 1	3:00 PM	2390.51	2060.78	1398.44	2710.35	800.75	1940.15	1308.45
8	Male	Day 1	3:00 PM	1846.65	1674.89	1279.05	2231.76	896.75	1576.10	1289.51
1	Female	Day 1	9:00 PM	2019.66	1837.90	1487.71	2715.62	1168.97	1731.24	1610.35
2	Female	Day 1	9:00 PM	2232.30	1855.76	1720.52	2667.41	1324.05	1836.52	1740.14
3	Female	Day 1	9:00 PM	2213.36	1881.90	1408.53	2743.37	1028.61	1829.75	1461.05
4	Female	Day 1	9:00 PM	1859.34	1624.48	1372.38	2561.43	1209.82	1770.83	1223.20
5	Male	Day 1	9:00 PM	2452.35	2159.32	1326.43	2536.22	1190.48	2166.62	1344.04
6	Male	Day 1	9:00 PM	1972.40	1862.78	1217.66	2454.36	893.94	1765.57	1389.88
7	Female	Day 1	9:00 PM	2398.30	2025.49	1411.27	2766.36	741.92	1956.20	1335.31
8	Male	Day 1	9:00 PM	1810.04	1680.82	1267.40	2221.05	927.55	1577.91	1251.19
1	Female	Day 2	9:00 AM	2064.27	1646.60	1494.98	2584.78	1114.01	1662.28	1627.58
2	Female	Day 2	9:00 AM	2064.36	1885.41	1675.27	2532.04	1232.98	1897.29	1760.60
3	Female	Day 2	9:00 AM	2172.58	1830.14	1363.34	2798.37	996.61	1711.90	1346.33
4	Female	Day 2	9:00 AM	1628.79	1664.31	1334.65	2713.88	1224.51	1666.08	1189.17
5	Male	Day 2	9:00 AM	2328.18	2048.25	1328.32	2413.47	1331.51	2122.90	1311.04
6	Male	Day 2	9:00 AM	1969.48	1832.38	1242.17	2427.32	944.00	1751.29	1328.91
7	Female	Day 2	9:00 AM	1903.52	1576.83	1386.86	2351.92	745.47	1473.25	1263.82
8	Male	Day 2	9:00 AM	1878.01	1688.58	1240.59	2261.15	969.58	1517.11	1249.41
1	Female	Day 2	3:00 PM	1988.86	1784.37	1398.12	2731.85	1106.38	1717.82	1539.95
2	Female	Day 2	3:00 PM	1980.34	1801.03	1707.39	2360.45	1384.84	1880.56	1711.12
3	Female	Day 2	3:00 PM	2189.94	1842.79	1376.20	2784.96	1022.48	1794.80	1337.81
4	Female	Day 2	3:00 PM	2092.80	1957.10	1363.93	2329.24	1309.27	1801.27	1185.22
5	Male	Day 2	3:00 PM	2318.47	2026.31	1278.26	2417.37	1927.39	2123.57	1317.59
6	Male	Day 2	3:00 PM	1940.65	1832.39	1272.87	2366.55	941.21	1678.37	1257.33
7	Female	Day 2	3:00 PM	2405.50	2017.00	1398.29	2665.11	706.83	1916.30	1325.64
8	Male	Day 2	3:00 PM	1860.71	1763.30	1244.60	2274.40	976.71	1590.26	1240.02
1	Female	Day 2	9:00 PM	1978.12	1781.49	1409.77	2632.60	1052.18	1784.09	1528.65
2	Female	Day 2	9:00 PM	1999.09	1818.29	1696.48	2240.22	1334.22	1836.27	1785.33
3	Female	Day 2	9:00 PM	2170.33	1849.48	1371.63	2695.40	1061.25	1817.27	1365.35
4	Female	Day 2	9:00 PM	2202.27	1977.28	1344.18	2181.96	1235.00	1846.52	1180.89
5	Male	Day 2	9:00 PM	2305.62	2065.04	1268.70	2470.60	2035.82	2171.64	1323.83
6	Male	Day 2	9:00 PM	1976.24	1836.14	1259.70	2412.77	906.74	1649.32	1228.78
7	Female	Day 2	9:00 PM	2292.62	1947.77	1414.85	2798.85	769.84	1826.94	1213.58
8	Male	Day 2	9:00 PM	1861.18	1759.73	1239.04	2288.33	940.51	1605.10	1228.78
1	Female	Day 3	9:00 AM	2085.65	1784.00	1408.36	2725.26	972.20	1737.06	1476.09
2	Female	Day 3	9:00 AM	2184.00	1923.06	1739.45	2217.93	1370.09	2086.07	1776.22
3	Female	Day 3	9:00 AM	2142.06	1810.63	1369.00	2718.53	1001.49	1743.35	1366.10
4	Female	Day 3	9:00 AM	1747.84	1786.87	1345.67	2504.19	1203.04	1836.54	1164.46
5	Male	Day 3	9:00 AM	2257.69	1955.76	1284.20	2318.02	1599.15	2042.40	1415.32
6	Male	Day 3	9:00 AM	1911.56	1759.73	1220.85	2388.27	949.63	1664.69	1209.29
7	Female	Day 3	9:00 AM	2378.39	2092.13	1412.40	2748.51	755.99	1857.44	1205.03
8	Male	Day 3	9:00 AM	1787.37	1571.71	1221.62	2148.47	973.83	1258.70	1199.78
1	Female	Day 3	3:00 PM	2032.38	1825.62	1487.91	2633.74	1170.33	1921.19	1572.67
2	Female	Day 3	3:00 PM	2199.12	1798.26	1685.10	2276.67	1334.59	1929.66	1748.11
3	Female	Day 3	3:00 PM	2242.85	1918.37	1391.84	2819.19	990.67	1817.70	1375.27
4	Female	Day 3	3:00 PM	1993.66	1781.09	1368.63	2418.80	1159.93	1675.46	1207.28
5	Male	Day 3	3:00 PM	2280.92	2040.09	1259.23	2425.15	1295.56	2152.49	1272.64
6	Male	Day 3	3:00 PM	1966.32	1838.45	1217.32	2379.05	915.40	1774.96	1245.31
7	Female	Day 3	3:00 PM	2439.60	1837.92	1414.03	2804.52	743.47	1903.40	1185.54
8	Male	Day 3	3:00 PM	1854.67	1730.38	1223.78	2227.70	920.54	1575.20	1210.26
1	Female	Day 3	9:00 PM	2034.68	1840.01	1403.75	2781.13	1059.61	1810.69	1463.95
2	Female	Day 3	9:00 PM	2096.18	1881.10	1754.94	1904.89	1372.30	1869.96	1738.55
3	Female	Day 3	9:00 PM	2178.71	1821.57	1367.44	2569.58	1000.54	1744.05	1361.88
4	Female	Day 3	9:00 PM	1405.67	1482.34	1390.67	2749.19	1226.33	1503.61	1199.39
5	Male	Day 3	9:00 PM	2330.29	2053.56	1203.09	2438.01	1400.01	2146.35	1203.61
6	Male	Day 3	9:00 PM	2027.21	1848.43	1229.40	2467.97	861.16	1742.73	1280.61
7	Female	Day 3	9:00 PM	2429.94	2029.14	1426.77	2854.61	787.01	1938.51	1201.49
8	Male	Day 3	9:00 PM	1830.37	1714.76	1227.24	2201.00	949.54	1571.94	1228.42
