# Supplementary material for: Variability in Vowel Production within and between Days
Source: PLoS One. 2015 Sep 2;10(9):e0136791. doi: 10.1371/journal.pone.0136791 (PMC4558024; doi:10.1371/journal.pone.0136791)
Supplement: S4 Table — (PDF) [file pone.0136791.s004.pdf]

Subject	Sex	Day	Time	Average F3 for /IH/	Average F3 for /EH/	Average F3 for /UH/	Average F3 for /EE/	Average F3 for /OO/	Average F3 for /AE/	Average F3 for /AH/
1	Female	Day 1	9:00 AM	3103.08	3061.28	3124.98	3374.20	3172.36	2938.09	2848.74
2	Female	Day 1	9:00 AM	2933.41	2807.98	3174.76	3409.66	2883.43	2584.25	2798.57
3	Female	Day 1	9:00 AM	2976.21	3005.57	3045.16	3239.11	2570.13	2862.13	2837.00
4	Female	Day 1	9:00 AM	2838.24	2788.74	3093.90	3137.26	2777.63	2638.56	3111.62
5	Male	Day 1	9:00 AM	2767.10	2706.20	2715.32	3191.59	2765.65	2548.54	2669.07
6	Male	Day 1	9:00 AM	2713.25	2650.43	2644.76	2924.77	2452.63	2667.73	2890.96
7	Female	Day 1	9:00 AM	3207.09	3074.47	3147.92	3511.55	2850.92	2823.27	2768.28
8	Male	Day 1	9:00 AM	2629.34	2418.23	2604.44	2732.13	2422.74	2330.35	2277.95
1	Female	Day 1	3:00 PM	3047.73	2698.93	2988.67	3184.31	3141.52	2450.40	2698.72
2	Female	Day 1	3:00 PM	2814.22	2650.10	3067.94	3381.61	2938.79	2526.19	2625.66
3	Female	Day 1	3:00 PM	3109.10	2981.42	3158.91	3314.67	2631.13	2922.60	2967.59
4	Female	Day 1	3:00 PM	2707.69	2825.89	3025.98	3192.75	2810.56	2818.81	3033.85
5	Male	Day 1	3:00 PM	2842.56	2763.41	2778.03	3082.38	2796.58	2683.55	2716.06
6	Male	Day 1	3:00 PM	2729.01	2684.14	2672.58	3023.13	2471.20	2763.52	2863.51
7	Female	Day 1	3:00 PM	3161.37	2975.11	3081.63	3509.46	2727.24	2695.51	2690.36
8	Male	Day 1	3:00 PM	2620.75	2506.01	2646.97	2756.30	2460.06	2222.42	2326.41
1	Female	Day 1	9:00 PM	2924.30	2847.73	2974.11	3264.49	3013.42	2777.98	2858.75
2	Female	Day 1	9:00 PM	3110.93	2903.67	3102.51	3475.55	3049.29	2689.23	2741.37
3	Female	Day 1	9:00 PM	3080.11	2920.35	3114.19	3305.08	2666.71	2912.94	2933.01
4	Female	Day 1	9:00 PM	2797.45	2557.53	3089.86	3233.13	2892.35	2755.31	3056.09
5	Male	Day 1	9:00 PM	2920.06	2771.20	2785.16	3217.34	2868.13	2734.10	2767.10
6	Male	Day 1	9:00 PM	2767.88	2650.92	2721.88	3210.86	2475.21	2749.45	2795.43
7	Female	Day 1	9:00 PM	3259.55	2824.32	3170.32	3707.19	2782.55	2729.99	2740.46
8	Male	Day 1	9:00 PM	2607.11	2580.11	2650.51	2715.78	2496.35	2209.36	2438.74
1	Female	Day 2	9:00 AM	3025.60	2698.95	3058.01	3158.41	3100.70	2701.36	2937.33
2	Female	Day 2	9:00 AM	2912.06	2775.38	3072.42	3443.90	2812.22	2725.53	2910.01
3	Female	Day 2	9:00 AM	3062.96	2913.42	3039.03	3303.41	2648.40	2865.42	2825.48
4	Female	Day 2	9:00 AM	2626.22	2626.41	3072.87	3181.24	2940.01	2636.30	3089.52
5	Male	Day 2	9:00 AM	2821.02	2680.32	2761.71	3050.31	2842.03	2676.48	2739.64
6	Male	Day 2	9:00 AM	2707.90	2653.37	2793.82	3091.41	2606.56	2683.02	2930.28
7	Female	Day 2	9:00 AM	2667.92	2397.11	3126.97	3489.41	2707.56	2169.69	2684.38
8	Male	Day 2	9:00 AM	2698.74	2564.10	2675.64	2801.28	2511.60	2382.20	2493.34
1	Female	Day 2	3:00 PM	2901.94	2836.71	2904.40	3200.83	2874.41	2550.20	2629.95
2	Female	Day 2	3:00 PM	2731.54	2665.99	3076.37	3260.60	2909.48	2679.24	2836.10
3	Female	Day 2	3:00 PM	3054.10	2995.67	3163.51	3296.20	2754.47	2898.46	2887.67
4	Female	Day 2	3:00 PM	2604.70	2659.57	3078.08	3025.57	2871.18	2509.76	3146.65
5	Male	Day 2	3:00 PM	2900.35	2695.02	2768.37	3097.25	3132.15	2745.61	2830.45
6	Male	Day 2	3:00 PM	2671.96	2601.45	2674.36	2966.89	2409.58	2572.08	2867.48
7	Female	Day 2	3:00 PM	3222.77	2742.98	3118.76	3785.50	2837.41	2658.60	2630.13
8	Male	Day 2	3:00 PM	2707.30	2658.29	2742.97	2847.10	2536.95	2521.32	2549.73
1	Female	Day 2	9:00 PM	2820.84	2773.59	2822.31	3018.54	2952.45	2442.95	2551.99
2	Female	Day 2	9:00 PM	2910.19	2702.75	3030.34	3382.08	2834.03	2596.10	2949.88
3	Female	Day 2	9:00 PM	3000.91	2880.00	3156.92	3131.12	2693.97	2861.70	2978.27
4	Female	Day 2	9:00 PM	2595.21	2568.54	3099.28	3103.91	2890.45	2491.05	3122.19
5	Male	Day 2	9:00 PM	2869.84	2702.52	2684.51	3072.72	3187.94	2739.52	2755.14
6	Male	Day 2	9:00 PM	2708.48	2660.58	2683.62	2987.01	2538.55	2637.94	2794.51
7	Female	Day 2	9:00 PM	3039.43	2599.04	3252.07	3736.46	2915.85	2364.57	2756.52
8	Male	Day 2	9:00 PM	2637.76	2595.41	2686.06	2846.14	2573.95	2413.65	2602.90
1	Female	Day 3	9:00 AM	3098.36	2753.44	3021.99	3280.46	3096.39	2790.92	2731.60
2	Female	Day 3	9:00 AM	3021.67	2823.57	3006.46	3354.32	2879.75	2938.72	2852.08
3	Female	Day 3	9:00 AM	2977.87	2918.76	3086.60	3251.60	2697.56	2801.95	2847.87
4	Female	Day 3	9:00 AM	2676.29	2785.43	3123.05	3122.63	2914.86	2806.72	3140.97
5	Male	Day 3	9:00 AM	2809.37	2604.13	2597.73	2959.21	2935.24	2627.63	2761.49
6	Male	Day 3	9:00 AM	2702.24	2611.50	2751.49	2980.90	2547.01	2751.52	2815.79
7	Female	Day 3	9:00 AM	3354.04	3152.38	3292.37	3686.34	2885.50	2768.27	2855.38
8	Male	Day 3	9:00 AM	2471.67	2281.88	2482.43	2657.65	2553.29	1922.91	2108.02
1	Female	Day 3	3:00 PM	3092.35	2984.18	3085.57	3230.81	3159.69	2975.05	2920.21
2	Female	Day 3	3:00 PM	2975.89	2409.21	2897.92	3256.85	2947.04	2628.67	2785.41
3	Female	Day 3	3:00 PM	3051.23	2903.40	3127.13	3262.72	2688.21	2808.05	3076.38
4	Female	Day 3	3:00 PM	2635.86	2544.19	3157.87	3091.21	2893.74	2441.14	3193.90
5	Male	Day 3	3:00 PM	2846.68	2759.29	2803.27	3062.44	2758.62	2711.16	2904.96
6	Male	Day 3	3:00 PM	2647.59	2670.91	2751.53	3039.22	2518.45	2700.54	2807.10
7	Female	Day 3	3:00 PM	3369.79	2815.19	3343.44	3812.07	2810.70	2673.66	2848.92
8	Male	Day 3	3:00 PM	2657.89	2567.48	2630.94	2820.78	2592.31	2265.88	2393.23
1	Female	Day 3	9:00 PM	2971.89	2912.68	2986.06	3267.90	3013.98	2678.92	2874.45
2	Female	Day 3	9:00 PM	2936.70	2787.15	2956.11	3262.09	2718.01	2727.93	2838.46
3	Female	Day 3	9:00 PM	2947.76	2756.91	3124.29	3144.32	2759.37	2720.39	2908.75
4	Female	Day 3	9:00 PM	2551.91	2357.61	3044.34	3193.95	2897.23	2476.18	3111.84
5	Male	Day 3	9:00 PM	2858.77	2672.52	2709.83	3091.72	2875.91	2671.05	2894.51
6	Male	Day 3	9:00 PM	2727.37	2691.46	2712.42	2974.31	2532.49	2695.88	2823.74
7	Female	Day 3	9:00 PM	3218.80	2909.24	3306.66	3844.46	2838.42	2741.66	2877.78
8	Male	Day 3	9:00 PM	2608.89	2550.87	2641.20	2873.54	2516.32	2442.43	2387.65
