# Supplementary material for: Variability in Vowel Production within and between Days
Source: PLoS One. 2015 Sep 2;10(9):e0136791. doi: 10.1371/journal.pone.0136791 (PMC4558024; doi:10.1371/journal.pone.0136791)
Supplement: S5 Table — (PDF) [file pone.0136791.s005.pdf]

Subject	Sex	Day	Time	Average SD in F0 for /IH/	Average SD in F0 for /EH/	Average SD in F0 for /UH/	Average SD in F0 for /EE/	Average SD in F0 for /OO/	Average SD in F0 for /AE/	Average SD in F0 for /AH/
1	Female	Day 1	9:00 AM	44.99	40.89	42.91	33.19	10.97	41.27	31.15
2	Female	Day 1	9:00 AM	3.67	3.50	4.23	39.68	40.58	3.44	37.41
3	Female	Day 1	9:00 AM	8.90	7.09	6.71	6.28	7.89	5.80	9.80
4	Female	Day 1	9:00 AM	8.01	9.27	8.92	6.91	13.34	5.73	9.43
5	Male	Day 1	9:00 AM	4.01	2.65	5.42	3.33	3.60	2.66	4.53
6	Male	Day 1	9:00 AM	14.91	14.79	12.74	14.67	16.47	14.94	15.82
7	Female	Day 1	9:00 AM	8.30	7.85	11.58	12.02	11.77	26.66	11.51
8	Male	Day 1	9:00 AM	3.93	20.24	4.20	4.34	4.52	4.40	3.68
1	Female	Day 1	3:00 PM	5.40	8.85	3.76	7.12	6.07	3.38	3.04
2	Female	Day 1	3:00 PM	50.52	39.34	9.67	6.37	5.64	6.91	11.58
3	Female	Day 1	3:00 PM	4.73	7.25	5.69	6.23	5.46	4.56	5.25
4	Female	Day 1	3:00 PM	4.07	8.72	8.94	6.98	4.73	6.04	5.15
5	Male	Day 1	3:00 PM	3.01	2.03	3.51	2.27	4.19	3.35	2.06
6	Male	Day 1	3:00 PM	3.53	2.81	3.56	2.80	3.49	9.75	2.94
7	Female	Day 1	3:00 PM	7.97	11.64	9.69	7.98	6.21	7.05	3.87
8	Male	Day 1	3:00 PM	2.92	3.17	1.92	2.20	2.66	1.10	1.90
1	Female	Day 1	9:00 PM	34.05	16.12	8.74	9.93	9.42	10.86	12.88
2	Female	Day 1	9:00 PM	39.63	5.57	8.04	3.96	5.16	5.49	8.42
3	Female	Day 1	9:00 PM	10.55	7.39	10.27	11.39	10.70	6.08	8.39
4	Female	Day 1	9:00 PM	7.58	3.25	11.44	5.80	5.79	6.98	4.89
5	Male	Day 1	9:00 PM	3.21	3.42	2.21	2.82	2.33	2.20	3.67
6	Male	Day 1	9:00 PM	3.34	4.27	56.96	6.21	3.80	4.43	25.66
7	Female	Day 1	9:00 PM	6.31	5.28	9.39	6.95	11.66	4.10	5.93
8	Male	Day 1	9:00 PM	3.00	2.75	2.28	2.67	2.06	3.85	2.31
1	Female	Day 2	9:00 AM	5.85	4.34	6.94	4.89	6.03	8.51	6.86
2	Female	Day 2	9:00 AM	4.83	15.85	2.97	38.62	39.86	40.15	2.99
3	Female	Day 2	9:00 AM	5.53	4.71	7.94	6.83	3.83	4.44	7.61
4	Female	Day 2	9:00 AM	6.75	5.32	6.93	4.69	6.01	4.66	7.54
5	Male	Day 2	9:00 AM	3.10	5.62	2.84	2.51	2.54	4.01	3.84
6	Male	Day 2	9:00 AM	3.77	2.17	1.76	2.45	2.80	2.85	9.73
7	Female	Day 2	9:00 AM	7.30	15.70	8.52	7.07	6.70	9.23	5.71
8	Male	Day 2	9:00 AM	1.78	1.98	1.62	1.81	2.31	1.48	2.34
1	Female	Day 2	3:00 PM	8.69	7.77	7.08	13.32	6.01	5.16	6.08
2	Female	Day 2	3:00 PM	8.44	5.04	4.50	11.98	40.92	5.85	4.17
3	Female	Day 2	3:00 PM	9.74	10.69	8.71	7.16	7.81	4.83	9.33
4	Female	Day 2	3:00 PM	4.57	3.70	5.54	6.99	4.35	3.68	4.48
5	Male	Day 2	3:00 PM	3.54	3.62	2.86	4.36	2.82	3.99	4.01
6	Male	Day 2	3:00 PM	3.25	2.93	2.36	2.55	2.23	2.18	5.72
7	Female	Day 2	3:00 PM	10.23	7.06	6.64	11.05	11.52	6.82	5.68
8	Male	Day 2	3:00 PM	2.32	1.53	1.28	1.70	11.43	2.36	1.71
1	Female	Day 2	9:00 PM	6.80	5.59	7.03	9.53	6.46	13.82	7.41
2	Female	Day 2	9:00 PM	4.43	16.31	5.97	6.75	3.95	7.40	4.87
3	Female	Day 2	9:00 PM	12.50	8.55	6.20	3.80	9.06	5.11	5.33
4	Female	Day 2	9:00 PM	3.91	5.41	3.65	2.79	3.13	3.26	3.08
5	Male	Day 2	9:00 PM	2.37	2.46	3.27	2.03	2.19	2.19	3.94
6	Male	Day 2	9:00 PM	2.67	2.81	2.51	2.69	3.06	2.14	2.61
7	Female	Day 2	9:00 PM	8.58	6.60	5.97	9.84	10.33	8.42	9.02
8	Male	Day 2	9:00 PM	1.79	1.85	1.83	1.56	2.40	1.63	1.85
1	Female	Day 3	9:00 AM	5.04	4.96	4.07	4.76	4.73	6.95	3.47
2	Female	Day 3	9:00 AM	4.35	7.70	6.55	7.26	6.77	4.93	4.91
3	Female	Day 3	9:00 AM	6.66	10.55	8.04	8.60	9.73	5.55	4.52
4	Female	Day 3	9:00 AM	4.65	5.75	5.92	7.10	4.95	7.37	6.10
5	Male	Day 3	9:00 AM	3.18	3.69	2.53	3.97	2.56	6.51	3.81
6	Male	Day 3	9:00 AM	2.94	3.12	2.54	5.32	1.37	2.29	1.46
7	Female	Day 3	9:00 AM	13.16	12.60	8.49	9.99	11.54	6.63	15.07
8	Male	Day 3	9:00 AM	1.45	1.34	1.37	1.39	0.90	1.67	1.34
1	Female	Day 3	3:00 PM	6.23	9.12	9.46	10.38	6.60	9.02	7.69
2	Female	Day 3	3:00 PM	6.14	5.01	6.33	7.38	7.00	5.72	3.37
3	Female	Day 3	3:00 PM	8.35	5.16	6.60	7.84	13.36	7.23	7.72
4	Female	Day 3	3:00 PM	6.93	4.31	5.80	5.40	57.99	15.75	6.28
5	Male	Day 3	3:00 PM	2.63	3.29	4.45	3.74	3.13	6.12	3.53
6	Male	Day 3	3:00 PM	2.36	4.73	5.58	3.94	5.73	3.38	3.66
7	Female	Day 3	3:00 PM	16.40	8.64	14.27	18.16	9.86	15.72	14.24
8	Male	Day 3	3:00 PM	1.25	1.93	1.68	1.30	2.42	1.35	1.17
1	Female	Day 3	9:00 PM	8.31	7.16	9.14	11.04	10.77	10.14	7.95
2	Female	Day 3	9:00 PM	15.94	12.88	39.01	5.42	5.39	7.81	29.91
3	Female	Day 3	9:00 PM	9.19	8.65	5.90	9.88	11.29	7.66	4.62
4	Female	Day 3	9:00 PM	2.32	6.18	9.30	2.81	8.36	12.36	5.82
5	Male	Day 3	9:00 PM	3.19	3.19	2.77	2.32	2.45	3.60	2.30
6	Male	Day 3	9:00 PM	42.24	3.16	7.96	5.33	8.19	2.93	2.82
7	Female	Day 3	9:00 PM	6.03	10.41	7.24	16.74	8.24	8.27	4.56
8	Male	Day 3	9:00 PM	1.56	1.56	1.99	1.30	3.95	2.90	1.92
