# Supplementary material for: Variability in Vowel Production within and between Days
Source: PLoS One. 2015 Sep 2;10(9):e0136791. doi: 10.1371/journal.pone.0136791 (PMC4558024; doi:10.1371/journal.pone.0136791)
Supplement: S6 Table — (PDF) [file pone.0136791.s006.pdf]

Subject	Sex	Day	Time	Average SD in F1 for /IH/	Average SD in F1 for /EH/	Average SD in F1 for /UH/	Average SD in F1 for /EE/	Average SD in F1 for /OO/	Average SD in F1 for /AE/	Average SD in F1 for /AH/
**1**	**Female**	**Day 1**	**9:00 AM**	30.16	25.26	34.09	18.00	13.14	49.33	48.96
**2**	**Female**	**Day 1**	**9:00 AM**	41.49	26.80	36.59	19.41	24.78	45.30	37.56
**3**	**Female**	**Day 1**	**9:00 AM**	22.47	18.09	63.92	13.71	7.99	28.42	25.13
**4**	**Female**	**Day 1**	**9:00 AM**	24.68	17.57	20.82	22.31	28.48	26.91	20.01
**5**	**Male**	**Day 1**	**9:00 AM**	17.05	15.70	15.45	5.55	45.32	39.48	19.13
**6**	**Male**	**Day 1**	**9:00 AM**	23.02	18.08	57.82	17.06	13.63	71.32	29.46
**7**	**Female**	**Day 1**	**9:00 AM**	37.90	41.25	124.86	13.25	15.92	31.51	63.59
**8**	**Male**	**Day 1**	**9:00 AM**	19.53	40.80	28.72	7.96	13.79	54.04	34.17
**1**	**Female**	**Day 1**	**3:00 PM**	25.14	24.60	25.07	32.50	27.25	36.87	34.98
**2**	**Female**	**Day 1**	**3:00 PM**	52.44	35.27	47.92	16.97	11.79	49.30	61.91
**3**	**Female**	**Day 1**	**3:00 PM**	21.02	16.25	46.87	12.32	16.44	24.16	25.74
**4**	**Female**	**Day 1**	**3:00 PM**	18.62	28.87	24.22	14.71	11.30	26.39	31.45
**5**	**Male**	**Day 1**	**3:00 PM**	12.40	16.57	42.97	3.94	45.96	35.17	23.70
**6**	**Male**	**Day 1**	**3:00 PM**	12.22	17.73	30.14	8.37	7.85	70.31	11.84
**7**	**Female**	**Day 1**	**3:00 PM**	20.29	42.33	41.46	9.99	16.72	35.23	47.29
**8**	**Male**	**Day 1**	**3:00 PM**	64.37	30.85	49.63	12.29	15.33	39.87	27.50
**1**	**Female**	**Day 1**	**9:00 PM**	46.71	23.08	27.55	24.11	22.04	36.74	44.87
**2**	**Female**	**Day 1**	**9:00 PM**	46.02	58.89	46.58	23.79	10.74	19.37	38.13
**3**	**Female**	**Day 1**	**9:00 PM**	20.60	28.14	34.48	13.97	15.19	17.88	31.66
**4**	**Female**	**Day 1**	**9:00 PM**	30.33	27.31	26.91	14.20	8.54	19.91	34.66
**5**	**Male**	**Day 1**	**9:00 PM**	19.23	22.31	28.59	4.87	57.80	27.48	14.75
**6**	**Male**	**Day 1**	**9:00 PM**	7.95	12.97	35.47	10.33	14.77	89.98	61.83
**7**	**Female**	**Day 1**	**9:00 PM**	16.73	27.33	51.36	7.55	12.99	20.37	53.52
**8**	**Male**	**Day 1**	**9:00 PM**	39.45	20.12	20.99	23.18	12.17	28.31	36.95
**1**	**Female**	**Day 2**	**9:00 AM**	35.63	15.48	23.98	14.55	15.39	59.32	36.74
**2**	**Female**	**Day 2**	**9:00 AM**	23.71	30.79	33.80	19.71	12.76	29.49	42.10
**3**	**Female**	**Day 2**	**9:00 AM**	34.78	23.97	23.15	11.33	7.40	15.39	11.44
**4**	**Female**	**Day 2**	**9:00 AM**	32.03	19.92	19.35	14.19	9.71	35.13	31.80
**5**	**Male**	**Day 2**	**9:00 AM**	12.94	18.37	69.62	113.27	62.75	23.81	23.25
**6**	**Male**	**Day 2**	**9:00 AM**	13.00	29.50	19.62	25.64	29.51	32.19	29.82
**7**	**Female**	**Day 2**	**9:00 AM**	34.72	46.28	34.58	8.90	9.74	34.45	31.92
**8**	**Male**	**Day 2**	**9:00 AM**	35.67	22.84	23.74	13.74	15.37	30.35	21.96
**1**	**Female**	**Day 2**	**3:00 PM**	34.24	33.47	19.14	18.45	17.08	34.73	39.35
**2**	**Female**	**Day 2**	**3:00 PM**	75.72	53.96	21.94	34.31	10.82	58.38	39.34
**3**	**Female**	**Day 2**	**3:00 PM**	20.75	35.89	28.72	12.54	19.33	23.63	18.95
**4**	**Female**	**Day 2**	**3:00 PM**	29.50	27.18	25.67	19.88	15.80	47.98	22.78
**5**	**Male**	**Day 2**	**3:00 PM**	4.03	9.98	36.78	10.37	63.95	23.34	25.41
**6**	**Male**	**Day 2**	**3:00 PM**	14.21	16.22	18.81	11.57	12.09	49.38	30.63
**7**	**Female**	**Day 2**	**3:00 PM**	19.17	47.59	55.21	27.46	13.32	24.79	56.46
**8**	**Male**	**Day 2**	**3:00 PM**	26.95	26.02	21.50	21.66	10.45	27.71	16.86
**1**	**Female**	**Day 2**	**9:00 PM**	15.74	37.70	40.75	21.35	22.54	23.42	33.07
**2**	**Female**	**Day 2**	**9:00 PM**	40.60	24.95	32.45	23.20	10.05	32.81	24.73
**3**	**Female**	**Day 2**	**9:00 PM**	26.85	37.02	37.79	8.63	13.17	17.38	21.43
**4**	**Female**	**Day 2**	**9:00 PM**	19.13	25.37	20.03	13.17	9.58	27.89	22.06
**5**	**Male**	**Day 2**	**9:00 PM**	20.81	19.60	27.49	7.51	56.54	30.54	15.20
**6**	**Male**	**Day 2**	**9:00 PM**	16.94	12.96	17.04	13.14	17.65	22.69	33.87
**7**	**Female**	**Day 2**	**9:00 PM**	23.84	58.66	37.03	21.40	9.78	37.20	57.43
**8**	**Male**	**Day 2**	**9:00 PM**	25.63	14.79	15.68	14.86	15.37	24.00	13.88
**1**	**Female**	**Day 3**	**9:00 AM**	47.98	32.72	23.15	38.85	14.23	48.07	33.52
**2**	**Female**	**Day 3**	**9:00 AM**	48.77	57.33	29.94	14.75	13.53	39.56	51.12
**3**	**Female**	**Day 3**	**9:00 AM**	38.88	17.25	19.25	15.82	16.38	26.54	23.56
**4**	**Female**	**Day 3**	**9:00 AM**	26.07	24.95	34.84	16.37	7.27	42.77	41.32
**5**	**Male**	**Day 3**	**9:00 AM**	23.83	57.06	90.37	93.77	31.84	39.89	78.24
**6**	**Male**	**Day 3**	**9:00 AM**	21.09	22.21	30.42	11.95	17.21	31.14	14.09
**7**	**Female**	**Day 3**	**9:00 AM**	40.59	157.68	57.96	27.71	15.90	27.46	31.87
**8**	**Male**	**Day 3**	**9:00 AM**	38.13	25.61	16.33	16.52	19.85	14.07	14.95
**1**	**Female**	**Day 3**	**3:00 PM**	37.80	18.31	36.16	35.64	23.23	46.33	36.25
**2**	**Female**	**Day 3**	**3:00 PM**	36.55	46.80	36.64	23.48	11.73	61.12	34.68
**3**	**Female**	**Day 3**	**3:00 PM**	29.42	16.45	21.76	11.25	12.25	11.91	13.66
**4**	**Female**	**Day 3**	**3:00 PM**	29.73	22.85	22.47	14.81	10.23	34.38	21.61
**5**	**Male**	**Day 3**	**3:00 PM**	11.42	16.22	30.14	5.96	25.03	23.42	27.71
**6**	**Male**	**Day 3**	**3:00 PM**	14.54	25.81	33.03	19.70	24.29	22.59	37.75
**7**	**Female**	**Day 3**	**3:00 PM**	43.46	32.72	23.11	21.69	10.14	31.56	52.27
**8**	**Male**	**Day 3**	**3:00 PM**	26.20	19.92	16.61	17.49	22.79	30.13	17.54
**1**	**Female**	**Day 3**	**9:00 PM**	31.49	36.32	39.16	22.83	14.73	44.11	23.88
**2**	**Female**	**Day 3**	**9:00 PM**	40.36	33.42	27.78	24.17	13.68	28.62	33.09
**3**	**Female**	**Day 3**	**9:00 PM**	26.71	15.79	30.33	15.44	19.99	30.88	26.96
**4**	**Female**	**Day 3**	**9:00 PM**	26.39	23.72	33.13	15.98	16.07	47.66	37.67
**5**	**Male**	**Day 3**	**9:00 PM**	12.54	17.03	25.62	7.00	26.75	25.52	22.85
**6**	**Male**	**Day 3**	**9:00 PM**	25.45	17.55	47.43	16.46	22.36	37.89	27.46
**7**	**Female**	**Day 3**	**9:00 PM**	40.55	32.89	33.54	17.86	33.13	18.41	31.95
**8**	**Male**	**Day 3**	**9:00 PM**	32.39	16.34	12.91	9.69	10.41	18.84	22.36
