# Supplementary material for: Variability in Vowel Production within and between Days
Source: PLoS One. 2015 Sep 2;10(9):e0136791. doi: 10.1371/journal.pone.0136791 (PMC4558024; doi:10.1371/journal.pone.0136791)
Supplement: S7 Table — (PDF) [file pone.0136791.s007.pdf]

Subject	Sex	Day	Time	Average SD in F2 for /IH/	Average SD in F2 for /EH/	Average SD in F2 for /UH/	Average SD in F2 for /EE/	Average SD in F2 for /OO/	Average SD in F2 for /AE/	Average SD in F2 for /AH/
**1**	**Female**	**Day 1**	**9:00 AM**	45.05	59.91	36.16	69.85	55.49	140.41	58.37
**2**	**Female**	**Day 1**	**9:00 AM**	203.15	157.30	50.45	309.21	110.15	185.84	91.31
**3**	**Female**	**Day 1**	**9:00 AM**	36.56	34.66	64.47	38.69	90.34	42.50	42.08
**4**	**Female**	**Day 1**	**9:00 AM**	274.63	226.42	41.60	324.80	64.26	92.25	40.73
**5**	**Male**	**Day 1**	**9:00 AM**	32.83	71.73	29.03	27.66	298.89	48.78	32.59
**6**	**Male**	**Day 1**	**9:00 AM**	75.10	26.88	44.40	52.01	32.14	65.18	39.64
**7**	**Female**	**Day 1**	**9:00 AM**	117.49	116.69	196.61	80.57	58.48	70.37	49.54
**8**	**Male**	**Day 1**	**9:00 AM**	35.74	51.44	41.98	56.35	74.49	80.45	18.39
**1**	**Female**	**Day 1**	**3:00 PM**	49.57	167.55	39.56	199.91	70.81	204.01	48.74
**2**	**Female**	**Day 1**	**3:00 PM**	207.26	158.06	50.65	367.86	57.86	115.68	116.08
**3**	**Female**	**Day 1**	**3:00 PM**	50.15	38.31	25.14	60.50	91.33	58.96	41.47
**4**	**Female**	**Day 1**	**3:00 PM**	213.90	266.03	44.06	370.31	64.58	89.38	33.37
**5**	**Male**	**Day 1**	**3:00 PM**	20.33	46.44	67.22	29.91	287.89	36.89	69.48
**6**	**Male**	**Day 1**	**3:00 PM**	42.38	52.89	22.02	39.87	35.60	159.03	25.33
**7**	**Female**	**Day 1**	**3:00 PM**	67.01	40.11	40.36	101.87	50.79	59.22	29.72
**8**	**Male**	**Day 1**	**3:00 PM**	139.03	64.08	50.56	74.80	51.32	68.39	57.54
**1**	**Female**	**Day 1**	**9:00 PM**	59.08	52.51	36.91	44.54	64.00	140.42	125.51
**2**	**Female**	**Day 1**	**9:00 PM**	150.83	242.77	62.70	505.47	153.19	225.37	71.96
**3**	**Female**	**Day 1**	**9:00 PM**	53.10	47.00	47.55	114.15	62.69	42.51	123.61
**4**	**Female**	**Day 1**	**9:00 PM**	253.84	148.48	34.04	262.30	40.07	175.39	35.24
**5**	**Male**	**Day 1**	**9:00 PM**	64.48	78.42	213.92	25.35	267.03	44.63	43.27
**6**	**Male**	**Day 1**	**9:00 PM**	69.12	34.45	23.29	26.57	33.80	98.85	229.81
**7**	**Female**	**Day 1**	**9:00 PM**	69.64	41.88	48.74	85.33	91.48	46.71	67.03
**8**	**Male**	**Day 1**	**9:00 PM**	17.55	48.61	28.53	69.19	48.09	55.05	20.08
**1**	**Female**	**Day 2**	**9:00 AM**	71.19	150.43	35.96	109.77	62.99	263.50	142.16
**2**	**Female**	**Day 2**	**9:00 AM**	183.00	174.55	16.48	366.34	50.29	148.45	28.78
**3**	**Female**	**Day 2**	**9:00 AM**	46.96	25.35	37.74	46.65	24.74	127.85	41.23
**4**	**Female**	**Day 2**	**9:00 AM**	190.93	159.49	22.99	81.86	48.16	215.25	41.05
**5**	**Male**	**Day 2**	**9:00 AM**	42.45	62.41	154.04	158.76	318.83	44.12	49.79
**6**	**Male**	**Day 2**	**9:00 AM**	51.88	60.03	21.84	18.69	109.92	23.07	50.24
**7**	**Female**	**Day 2**	**9:00 AM**	220.44	185.73	33.55	378.67	41.09	239.07	44.10
**8**	**Male**	**Day 2**	**9:00 AM**	54.00	48.77	46.89	39.62	60.45	79.19	40.16
**1**	**Female**	**Day 2**	**3:00 PM**	65.47	137.31	34.26	47.64	95.44	181.13	78.80
**2**	**Female**	**Day 2**	**3:00 PM**	263.12	246.70	54.84	320.06	177.91	113.34	72.00
**3**	**Female**	**Day 2**	**3:00 PM**	51.71	46.68	33.68	35.75	111.72	56.80	34.85
**4**	**Female**	**Day 2**	**3:00 PM**	147.05	66.82	31.70	198.66	62.78	187.12	47.63
**5**	**Male**	**Day 2**	**3:00 PM**	27.87	40.80	53.18	34.75	340.68	35.97	51.31
**6**	**Male**	**Day 2**	**3:00 PM**	32.95	25.89	28.38	23.71	46.50	55.48	38.31
**7**	**Female**	**Day 2**	**3:00 PM**	46.59	85.81	44.58	159.27	71.70	54.09	165.48
**8**	**Male**	**Day 2**	**3:00 PM**	34.73	52.17	40.77	46.89	21.12	30.71	24.34
**1**	**Female**	**Day 2**	**9:00 PM**	39.25	90.33	25.17	145.05	118.97	95.78	22.08
**2**	**Female**	**Day 2**	**9:00 PM**	159.49	141.12	34.56	414.59	83.71	150.85	45.63
**3**	**Female**	**Day 2**	**9:00 PM**	92.43	36.88	19.98	81.03	30.64	35.29	19.84
**4**	**Female**	**Day 2**	**9:00 PM**	125.29	255.79	30.59	222.60	60.88	213.06	34.85
**5**	**Male**	**Day 2**	**9:00 PM**	53.60	83.99	36.26	42.55	251.28	39.46	30.28
**6**	**Male**	**Day 2**	**9:00 PM**	54.81	41.92	21.03	21.14	42.06	41.84	50.68
**7**	**Female**	**Day 2**	**9:00 PM**	88.46	121.26	47.14	238.88	107.56	110.42	44.11
**8**	**Male**	**Day 2**	**9:00 PM**	23.93	46.55	25.27	36.85	50.83	29.73	22.89
**1**	**Female**	**Day 3**	**9:00 AM**	65.75	109.64	44.61	75.13	43.42	246.26	57.84
**2**	**Female**	**Day 3**	**9:00 AM**	198.85	201.58	41.61	362.30	97.82	75.86	46.36
**3**	**Female**	**Day 3**	**9:00 AM**	95.74	44.20	20.31	57.98	62.33	48.67	41.87
**4**	**Female**	**Day 3**	**9:00 AM**	220.18	180.66	43.10	343.52	56.11	162.24	36.37
**5**	**Male**	**Day 3**	**9:00 AM**	60.13	81.54	49.50	186.99	248.19	65.64	254.62
**6**	**Male**	**Day 3**	**9:00 AM**	48.88	53.25	31.95	61.43	86.85	37.51	39.59
**7**	**Female**	**Day 3**	**9:00 AM**	60.18	240.98	36.49	137.70	48.41	106.99	47.86
**8**	**Male**	**Day 3**	**9:00 AM**	97.29	119.29	24.51	92.84	48.21	90.07	54.72
**1**	**Female**	**Day 3**	**3:00 PM**	94.30	52.90	36.33	78.01	108.01	86.88	44.47
**2**	**Female**	**Day 3**	**3:00 PM**	177.08	122.64	50.55	417.18	92.25	92.17	43.22
**3**	**Female**	**Day 3**	**3:00 PM**	58.05	40.26	40.86	79.44	88.73	55.46	39.19
**4**	**Female**	**Day 3**	**3:00 PM**	163.49	99.32	14.12	386.31	65.83	166.33	27.31
**5**	**Male**	**Day 3**	**3:00 PM**	83.04	57.97	105.16	25.36	255.62	43.09	64.20
**6**	**Male**	**Day 3**	**3:00 PM**	47.40	41.50	17.73	81.47	139.57	46.63	50.97
**7**	**Female**	**Day 3**	**3:00 PM**	78.75	209.52	62.01	128.50	102.77	107.99	71.79
**8**	**Male**	**Day 3**	**3:00 PM**	34.78	31.05	22.28	35.40	47.91	39.60	37.33
**1**	**Female**	**Day 3**	**9:00 PM**	57.67	87.81	38.72	48.95	111.57	197.39	65.92
**2**	**Female**	**Day 3**	**9:00 PM**	125.82	138.54	60.40	412.49	73.19	91.69	50.17
**3**	**Female**	**Day 3**	**9:00 PM**	76.39	52.08	24.12	263.98	77.53	96.30	52.38
**4**	**Female**	**Day 3**	**9:00 PM**	166.06	264.53	25.46	128.73	66.09	218.26	57.38
**5**	**Male**	**Day 3**	**9:00 PM**	44.61	73.88	21.02	33.28	255.44	36.32	37.59
**6**	**Male**	**Day 3**	**9:00 PM**	41.97	42.72	24.47	47.25	97.15	34.48	67.24
**7**	**Female**	**Day 3**	**9:00 PM**	53.56	144.97	38.54	121.47	59.58	135.40	38.02
**8**	**Male**	**Day 3**	**9:00 PM**	52.02	37.51	21.24	35.53	57.93	32.18	29.50
