# Supplementary material for: Variability in Vowel Production within and between Days
Source: PLoS One. 2015 Sep 2;10(9):e0136791. doi: 10.1371/journal.pone.0136791 (PMC4558024; doi:10.1371/journal.pone.0136791)
Supplement: S8 Table — (PDF) [file pone.0136791.s008.pdf]

Subject	Sex	Day	Time	Average SD in F3 for /IH/	Average SD in F3 for /EH/	Average SD in F3 for /UH/	Average SD in F3 for /EE/	Average SD in F3 for /OO/	Average SD in F3 for /AE/	Average SD in F3 for /AH/
1	Female	Day 1	9:00 AM	38.36	108.34	90.04	30.12	131.85	233.57	234.07
2	Female	Day 1	9:00 AM	273.37	259.82	66.73	145.10	90.07	154.31	146.94
3	Female	Day 1	9:00 AM	243.35	131.17	172.15	174.24	191.39	165.03	186.51
4	Female	Day 1	9:00 AM	305.52	290.87	66.98	111.84	64.33	252.57	60.49
5	Male	Day 1	9:00 AM	29.27	93.81	48.13	58.32	70.86	32.20	40.67
6	Male	Day 1	9:00 AM	49.18	117.36	101.77	85.35	66.73	233.88	190.98
7	Female	Day 1	9:00 AM	214.92	165.81	149.32	181.49	160.29	239.89	115.51
8	Male	Day 1	9:00 AM	83.66	129.05	128.25	67.48	74.65	187.91	194.90
1	Female	Day 1	3:00 PM	110.37	277.49	182.86	102.83	63.42	304.89	267.73
2	Female	Day 1	3:00 PM	164.03	193.91	177.80	93.92	116.34	76.45	289.74
3	Female	Day 1	3:00 PM	42.22	95.54	96.28	76.89	79.07	76.54	129.73
4	Female	Day 1	3:00 PM	158.24	345.17	109.21	92.20	71.05	179.62	79.09
5	Male	Day 1	3:00 PM	26.35	67.15	46.76	114.43	58.34	71.66	43.47
6	Male	Day 1	3:00 PM	56.96	48.54	49.73	91.92	40.00	150.57	205.58
7	Female	Day 1	3:00 PM	97.38	184.58	92.56	221.90	168.27	248.65	69.30
8	Male	Day 1	3:00 PM	97.84	113.30	83.93	69.32	106.84	118.18	178.70
1	Female	Day 1	9:00 PM	97.12	125.24	63.83	59.72	147.79	131.58	66.51
2	Female	Day 1	9:00 PM	147.11	227.03	154.88	155.72	128.85	264.04	278.69
3	Female	Day 1	9:00 PM	57.75	89.29	65.79	85.32	50.77	86.44	98.45
4	Female	Day 1	9:00 PM	171.13	233.59	57.36	88.72	54.66	220.96	79.20
5	Male	Day 1	9:00 PM	63.71	67.45	19.85	135.94	99.18	41.73	45.13
6	Male	Day 1	9:00 PM	60.60	49.42	83.16	133.62	24.17	175.38	156.32
7	Female	Day 1	9:00 PM	105.84	222.56	82.12	179.28	107.17	199.50	79.90
8	Male	Day 1	9:00 PM	90.74	92.50	47.32	77.35	128.92	134.73	94.54
1	Female	Day 2	9:00 AM	67.16	282.74	106.45	63.90	95.90	347.45	202.51
2	Female	Day 2	9:00 AM	147.26	224.14	53.58	139.01	140.71	163.96	64.28
3	Female	Day 2	9:00 AM	30.75	99.65	93.55	63.07	60.78	54.60	60.91
4	Female	Day 2	9:00 AM	112.92	181.13	81.54	61.12	38.23	222.62	73.73
5	Male	Day 2	9:00 AM	45.83	28.45	96.58	156.64	81.17	28.94	43.79
6	Male	Day 2	9:00 AM	37.09	89.30	43.88	158.75	40.13	131.37	103.87
7	Female	Day 2	9:00 AM	82.20	189.52	81.73	123.93	151.43	247.73	90.14
8	Male	Day 2	9:00 AM	32.20	159.89	68.30	39.50	130.12	208.93	102.45
1	Female	Day 2	3:00 PM	134.15	239.41	61.54	85.63	76.37	215.45	234.26
2	Female	Day 2	3:00 PM	258.83	217.97	63.20	108.32	89.88	151.31	99.13
3	Female	Day 2	3:00 PM	96.29	85.65	81.07	100.31	52.71	90.06	67.17
4	Female	Day 2	3:00 PM	175.01	237.41	56.24	86.67	64.66	363.77	40.73
5	Male	Day 2	3:00 PM	44.03	33.93	26.58	68.84	162.54	57.58	26.78
6	Male	Day 2	3:00 PM	46.48	53.35	63.72	53.29	24.39	90.28	57.60
7	Female	Day 2	3:00 PM	124.36	259.51	104.63	175.25	81.77	153.25	95.95
8	Male	Day 2	3:00 PM	29.48	80.55	29.60	59.55	74.13	127.05	86.09
1	Female	Day 2	9:00 PM	93.38	225.61	77.09	85.66	80.76	201.30	148.59
2	Female	Day 2	9:00 PM	143.17	185.37	115.52	127.48	109.58	120.10	65.06
3	Female	Day 2	9:00 PM	60.51	85.69	41.00	127.16	99.87	39.43	66.94
4	Female	Day 2	9:00 PM	96.34	231.51	45.57	96.69	83.81	291.34	71.00
5	Male	Day 2	9:00 PM	67.33	20.20	17.05	64.51	241.07	91.28	29.80
6	Male	Day 2	9:00 PM	31.79	43.35	39.06	108.03	32.83	84.80	133.94
7	Female	Day 2	9:00 PM	188.14	334.62	53.47	86.72	121.47	181.53	140.31
8	Male	Day 2	9:00 PM	65.57	89.51	61.68	84.55	78.52	157.76	93.93
1	Female	Day 3	9:00 AM	110.79	359.40	149.74	122.12	103.48	396.66	293.62
2	Female	Day 3	9:00 AM	249.82	365.47	73.37	75.90	176.23	145.32	253.54
3	Female	Day 3	9:00 AM	118.11	70.82	73.08	88.89	77.90	84.39	121.00
4	Female	Day 3	9:00 AM	177.95	283.82	31.04	76.47	60.21	202.10	104.91
5	Male	Day 3	9:00 AM	65.67	69.53	46.10	160.84	67.51	31.90	122.16
6	Male	Day 3	9:00 AM	62.13	69.57	50.98	110.74	105.43	72.31	138.84
7	Female	Day 3	9:00 AM	90.62	99.55	89.42	144.18	128.27	244.29	131.57
8	Male	Day 3	9:00 AM	143.26	214.89	141.89	103.08	121.16	102.68	166.60
1	Female	Day 3	3:00 PM	100.72	107.22	128.21	50.15	120.20	288.90	116.13
2	Female	Day 3	3:00 PM	159.93	140.21	204.21	181.25	134.70	162.90	190.80
3	Female	Day 3	3:00 PM	122.28	95.18	74.16	77.88	91.44	110.58	65.18
4	Female	Day 3	3:00 PM	237.06	269.01	121.44	99.37	30.14	262.66	67.33
5	Male	Day 3	3:00 PM	88.95	51.35	41.58	69.55	94.90	21.30	22.61
6	Male	Day 3	3:00 PM	36.16	113.93	68.32	73.87	58.20	132.02	98.29
7	Female	Day 3	3:00 PM	92.13	234.54	73.35	164.09	118.78	329.56	226.07
8	Male	Day 3	3:00 PM	93.91	139.26	98.75	44.08	65.92	161.39	31.48
1	Female	Day 3	9:00 PM	80.85	217.32	91.39	102.54	99.52	222.52	177.96
2	Female	Day 3	9:00 PM	159.72	258.96	97.64	68.52	154.90	156.07	176.49
3	Female	Day 3	9:00 PM	151.63	148.65	72.38	109.04	39.37	121.56	118.93
4	Female	Day 3	9:00 PM	102.62	192.29	68.40	77.86	48.21	211.06	37.32
5	Male	Day 3	9:00 PM	64.81	55.51	22.07	57.10	44.26	85.27	40.74
6	Male	Day 3	9:00 PM	48.75	45.39	43.71	117.50	65.58	54.32	151.66
7	Female	Day 3	9:00 PM	243.05	341.85	63.81	75.66	121.38	255.46	185.50
8	Male	Day 3	9:00 PM	80.97	114.81	56.69	67.93	124.45	124.45	130.37
