# Supplementary material for: Variability in Vowel Production within and between Days
Source: PLoS One. 2015 Sep 2;10(9):e0136791. doi: 10.1371/journal.pone.0136791 (PMC4558024; doi:10.1371/journal.pone.0136791)
Supplement: S9 Table — (PDF) [file pone.0136791.s009.pdf]

Subject	Sex	Day	Time	Average length for /IH/	Average length for /EH/	Average length for /UH/	Average length for /EE/	Average length for /OO/	Average length for /AE/	Average length for /AH/
1	**Female**	**Day 1**	**9:00 AM**	398	413	440	459	460	488	491
2	**Female**	**Day 1**	**9:00 AM**	172	194	182	214	213	218	219
3	**Female**	**Day 1**	**9:00 AM**	274	276	288	273	309	300	278
4	**Female**	**Day 1**	**9:00 AM**	220	219	220	234	257	229	237
5	**Male**	**Day 1**	**9:00 AM**	161	160	173	194	186	175	163
6	**Male**	**Day 1**	**9:00 AM**	164	213	217	222	214	212	232
7	**Female**	**Day 1**	**9:00 AM**	176	175	166	228	217	195	204
8	**Male**	**Day 1**	**9:00 AM**	250	268	290	291	299	276	300
1	**Female**	**Day 1**	**3:00 PM**	429	438	452	457	461	489	485
2	**Female**	**Day 1**	**3:00 PM**	192	194	214	245	230	239	229
3	**Female**	**Day 1**	**3:00 PM**	187	204	250	283	303	314	296
4	**Female**	**Day 1**	**3:00 PM**	232	211	236	237	247	241	227
5	**Male**	**Day 1**	**3:00 PM**	240	238	243	268	259	249	236
6	**Male**	**Day 1**	**3:00 PM**	213	235	252	253	241	229	256
7	**Female**	**Day 1**	**3:00 PM**	257	257	247	306	287	246	280
8	**Male**	**Day 1**	**3:00 PM**	217	223	237	234	229	250	254
1	**Female**	**Day 1**	**9:00 PM**	414	438	422	447	460	470	467
2	**Female**	**Day 1**	**9:00 PM**	176	183	206	223	211	218	216
3	**Female**	**Day 1**	**9:00 PM**	188	207	226	285	301	277	293
4	**Female**	**Day 1**	**9:00 PM**	254	249	257	252	262	264	266
5	**Male**	**Day 1**	**9:00 PM**	305	301	301	321	319	302	286
6	**Male**	**Day 1**	**9:00 PM**	188	211	219	217	223	194	214
7	**Female**	**Day 1**	**9:00 PM**	225	234	238	278	259	272	262
8	**Male**	**Day 1**	**9:00 PM**	265	270	290	290	265	290	304
1	**Female**	**Day 2**	**9:00 AM**	430	435	447	470	460	461	475
2	**Female**	**Day 2**	**9:00 AM**	172	170	181	196	206	211	206
3	**Female**	**Day 2**	**9:00 AM**	174	192	245	319	314	319	315
4	**Female**	**Day 2**	**9:00 AM**	234	231	228	241	259	236	240
5	**Male**	**Day 2**	**9:00 AM**	336	358	340	363	379	354	351
6	**Male**	**Day 2**	**9:00 AM**	198	217	239	230	245	224	243
7	**Female**	**Day 2**	**9:00 AM**	263	269	266	305	292	260	247
8	**Male**	**Day 2**	**9:00 AM**	330	331	342	353	301	360	373
1	**Female**	**Day 2**	**3:00 PM**	317	338	320	354	351	376	355
2	**Female**	**Day 2**	**3:00 PM**	170	179	200	203	211	210	213
3	**Female**	**Day 2**	**3:00 PM**	172	180	220	296	306	286	283
4	**Female**	**Day 2**	**3:00 PM**	206	196	194	216	206	211	206
5	**Male**	**Day 2**	**3:00 PM**	351	350	355	390	389	379	343
6	**Male**	**Day 2**	**3:00 PM**	191	212	224	214	221	214	222
7	**Female**	**Day 2**	**3:00 PM**	217	220	231	282	252	244	248
8	**Male**	**Day 2**	**3:00 PM**	434	461	458	462	467	497	482
1	**Female**	**Day 2**	**9:00 PM**	307	316	325	342	355	368	364
2	**Female**	**Day 2**	**9:00 PM**	182	193	213	218	226	236	229
3	**Female**	**Day 2**	**9:00 PM**	189	194	248	270	296	306	302
4	**Female**	**Day 2**	**9:00 PM**	205	198	209	209	211	215	221
5	**Male**	**Day 2**	**9:00 PM**	364	375	370	405	393	398	321
6	**Male**	**Day 2**	**9:00 PM**	184	198	214	198	210	198	208
7	**Female**	**Day 2**	**9:00 PM**	237	237	241	267	274	256	274
8	**Male**	**Day 2**	**9:00 PM**	405	400	415	425	422	415	423
1	**Female**	**Day 3**	**9:00 AM**	387	404	408	440	424	434	431
2	**Female**	**Day 3**	**9:00 AM**	191	193	208	228	228	228	224
3	**Female**	**Day 3**	**9:00 AM**	191	197	251	298	316	317	303
4	**Female**	**Day 3**	**9:00 AM**	244	235	234	238	259	239	245
5	**Male**	**Day 3**	**9:00 AM**	371	392	419	425	421	396	355
6	**Male**	**Day 3**	**9:00 AM**	208	227	246	221	250	234	244
7	**Female**	**Day 3**	**9:00 AM**	247	181	254	349	332	293	264
8	**Male**	**Day 3**	**9:00 AM**	538	522	560	555	539	536	537
1	**Female**	**Day 3**	**3:00 PM**	406	423	407	435	439	437	436
2	**Female**	**Day 3**	**3:00 PM**	196	197	218	235	236	243	237
3	**Female**	**Day 3**	**3:00 PM**	163	176	216	276	274	301	290
4	**Female**	**Day 3**	**3:00 PM**	194	202	198	203	206	193	215
5	**Male**	**Day 3**	**3:00 PM**	331	323	344	362	363	354	313
6	**Male**	**Day 3**	**3:00 PM**	186	186	190	175	206	194	205
7	**Female**	**Day 3**	**3:00 PM**	239	235	285	338	340	272	296
8	**Male**	**Day 3**	**3:00 PM**	554	554	535	576	577	568	573
1	**Female**	**Day 3**	**9:00 PM**	316	323	320	351	350	354	355
2	**Female**	**Day 3**	**9:00 PM**	207	222	238	244	243	252	252
3	**Female**	**Day 3**	**9:00 PM**	165	171	211	260	292	261	265
4	**Female**	**Day 3**	**9:00 PM**	237	225	235	238	240	243	255
5	**Male**	**Day 3**	**9:00 PM**	379	361	397	446	441	405	371
6	**Male**	**Day 3**	**9:00 PM**	158	170	162	171	184	161	168
7	**Female**	**Day 3**	**9:00 PM**	241	247	233	332	303	280	303
8	**Male**	**Day 3**	**9:00 PM**	569	554	596	609	584	601	596
